# Effectiveness of physical activity interventions on reducing perceived fatigue among adults with chronic conditions: a systematic review and meta-analysis of randomised controlled trials

**DOI:** 10.1038/s41598-023-41075-8

**Published:** 2023-09-04

**Authors:** Ioulia Barakou, Kandianos Emmanouil Sakalidis, Ulric Sena Abonie, Tracy Finch, Katie L. Hackett, Florentina Johanna Hettinga

**Affiliations:** 1 Department of Nursing, Midwifery & Health, Northumbria University, Newcastle upon Tyne, NE7 7XA UK; 2 Department of Sport Exercise and Rehabilitation, Faculty of Health and Life Sciences, Northumberland Building, Northumbria University, Newcastle upon Tyne, NE1 8ST UK; 3 Department of Social Work, Education and Community Wellbeing, Northumbria University, Newcastle upon Tyne, UK; 4 CRESTA Fatigue Clinic, Newcastle upon Tyne Hospitals NHS Foundation Trust, Newcastle upon Tyne, UK

**Keywords:** Diseases, Health care

## Abstract

Fatigue is barrier of physical activity participation in adults with chronic conditions. However, physical activity alleviates fatigue symptoms. This systematic review and meta-analysis aimed to (1) synthesise evidence from randomised controlled trials (RCTs) exploring the effects of physical activity interventions on fatigue reduction and (2) evaluate their effectiveness. Medline/CINAHL/EMBASE/Web of Science and Scopus were searched up to June 24th, 2023. Two reviewers independently conducted study screening and selection (RCTs), extracted data and assessed risk of bias (RoB2). Outcome was the standardised mean difference (SMD) with 95% confidence intervals in fatigue between experimental and control groups. 38 articles met the inclusion criteria. Overall, physical activity interventions moderately reduced fatigue (SMD = 0.70 *p* < 0.0001). Interventions lasting 2–6 weeks and 16–24 weeks demonstrated the larger effects on fatigue reductions (SMD=0.86, *p*<0.00001; SMD=1.82, *p*=0.01, respectively). Interventions with 30-36 sessions showed a large effect on fatigue reduction (SMD = 0.94, *p* < 0.04). Resistance, aerobic cycling and combination training interventions had a large to moderate effect (SMD= 0.93, *p* 0.03; SMD = 0.66, *p*= 0.0005; SMD = 0.76, *p* = <0.00001, respectively). Small long-term effects were found during follow-up(SMD=0.38, p= 0.002). Notably, both short (2-6 weeks) and longer-term (16-24 weeks) interventions were effective in reducing fatigue. . Physical activity interventions moderately reduced fatigue among adults with chronic conditions. Duration, total sessions, and mode of physical activity were identified as key factors in intervention effectiveness. Further research is needed to explore the impact of physical activity interventions on fatigue.

## Introduction

The prevalence of chronic conditions rises with age, with approximately 62% of Americans aged 65 and above having at least one chronic condition^1–3^. Fatigue is a common and complex symptom reported by individuals with various chronic conditions (e.g., cancer, Parkinson’s Disease, inflammatory arthritis, and fibromyalgia)^4–7^. Despite its common occurrence, fatigue lacks a clear definition due to its multidimensionality, encompassing physical, mental, cognitive, emotional, and motivational fatigue^8–10^. Within the literature, fatigue is described as a disruptive, severe, and overwhelming symptom with cognitive elements among adults with chronic conditions^8,11–14^. The prevalence of fatigue in this population ranges from 39 to 80%^6,15–18^, making it a significant factor associated with limitations in functional independence and a barrier to engaging in physical activity (PA) for individuals with chronic conditions experiencing fatigue symptoms^19–21^.

Furthermore, fatigue often co-exists with other medical symptoms, such as pain, depression, and cognitive deficit^22^. Also, significant fatigue can have a detrimental effect on daily activities and health-related quality of life among individuals with chronic conditions^19,23–25^. In daily life context, fatigue exhibits multiple negative effects, which have been observed to progressively escalate from impaired attention and reduced PA levels to increased risk of falls, disabling conditions, and mortality^9,26^. In general, fatigue has a negative impact on health and functioning of individuals with chronic conditions, leading to a decrease in their health-related quality of life^27^. Yet, the experience of fatigue symptoms often correlates with lower exercise engagement and a lack of independence^28,29^.

Lack of PA engagement is prevalent among adults with chronic conditions across the lifespan^19^. Fatigue can act as a barrier to activity engagement, as intense exercise during periods of fatigue can cause a negative affective load, further discouraging future engagement in activities^30,31^. However, the relationship between PA and fatigue is complex, with conflicting outcomes suggesting both positive effects of PA on fatigue reduction and the negative impact of fatigue on PA participation^9,20^. While some recommendations suggest that PA might reduce fatigue symptoms^32–35^, other studies indicate limited or no effects of exercise therapy on fatigue among adults with chronic fatigue syndrome^36^ and Parkinson’s disease^37^. Nevertheless, PA offers benefits for overall functioning, health, well-being, and quality of life and is associated with a reduced risk of premature mortality^13,38–42^. Specifically, exercise and PA interventions in adults with mild cognitive impairments have shown positive effects on cognitive function^40,43^. Additionally, resistance training has also demonstrated favorable outcomes in measures of reasoning^44^ and exergaming has been found to improve cognitive function^45^. Furthermore, PA has proven mental health benefits, reducing morbidity and mortality and promoting improved sleep^42,46^.

PA is a critical element of fatigue management and has the potential to positively impact individuals’ well-being and quality of life^47^. While exercise-based interventions can lead to notable improvements in fitness within few weeks, the challenge lies in sustaining these outcomes over time. For this reason, the effectiveness of these interventions may rely on various intervention ingredients, including the duration, total sessions, and mode of PA. These ingredients are important considerations for both health-professionals and end-users, with implications for cost-effectiveness and sustained outcomes^48^. For instance, a study on adolescents found that an intervention of 7 weeks is adequate for improving physical fitness^49^. Therefore, further exploration of the importance of intervention duration and the role of different PA modes in addressing fatigue is crucial. Moreover, the mode of PA might play a key role on PA interventions and the long-term effects of it. It has been found that people are more likely to participate in PA if they are enjoying it^50^, which might be related to preferred exercise mode.

In the view of the above, it is important to explore how PA interventions reduce fatigue symptoms in adults with chronic conditions. PA interventions targeting fatigue often focus on one specific chronic condition rather than addressing the symptom itself, despite its prevalence in various chronic conditions. Interventions targeting fatigue are essential in the onset of symptoms and could align with individuals’ needs^51^. Therefore, gaining a better understanding of fatigue transdiagnostically, could provide valuable insights into the relationship between PA and fatigue. Taking a transdiagnostic approach in studying fatigue could offer a broader perspective and enhance our understanding of effective fatigue management strategies. The transdiagnostic approach involves examining fatigue across multiple chronic conditions, considering that experiencing a chronic condition is frequently associated with fatigue^52,53^. Targeting fatigue management early in its onset through PA, may yield beneficial outcomes, particularly in cases where the underlying causes remain undetermined.

In the current review, we adopt a transdiagnostic approach, focusing on diverse chronic conditions. This approach is valuable and innovative as it emphasizes the importance of symptom-dependency and disease-independency. Notably, the existing literature lacks comprehensive reviews encompassing a wide range of chronic conditions, including both oncological and non-oncological conditions, regarding the potential of PA in reducing fatigue symptoms. Furthermore, this review explores various ingredients of PA interventions (intervention length, total sessions, mode of PA) and their impact on perceived fatigue. To the best of our knowledge, there are currently no published systematic reviews/meta-analyses primarily investigating the effectiveness of PA intervention ingredients in reducing fatigue among adults with various chronic conditions. The outcomes of this review could offer novel insights into a transdiagnostic approach that targets fatigue symptoms and underscores the importance of PA for individuals with chronic conditions. Additionally, this systematic review and meta-analysis may inform researchers and health-professionals about the further development of PA interventions, while recognising the need for additional research in this area. The selection of randomised controlled trials (RCTs) for inclusion in this systematic review is based on their recognised status as the highest quality evidence in evaluating interventions^54^. Though, we also appreciate other research methodologies and designs, RCTs provide robust evidence regarding the effectiveness of interventions and their results can inform the design of future interventions^55^. Overall, the high-quality evidence derived from RCTs is valuable in guiding clinical practice and decision-making^55^.

Based on the aforementioned rationale, the first aim of this review was to comprehensively synthesise evidence from RCTs on whether physical activity interventions significantly reduced fatigue symptoms among adults with chronic conditions. The second objective was to evaluate the effectiveness of PA interventions on perceived fatigue through a meta-analysis of the scientific literature.

## Methods

### Search strategy

The systematic review and meta-analysis were designed following the PRISMA statement for systematic review protocols and reporting guidelines^56^. The review was not registered. The search strategy was developed and tested in Medline using a combination of fatigue, PA, chronic disorder, and fatigue assessment terms. The search was modified for the following electronic databases: EMBASE; Web of Science; CINAHL; Scopus. No date limitations were applied. The search was performed on June 24th 2023. The full search strategy for all databases can be found in Table 1.

**Table 1:** Search strategy for all databases including Boolean operators for each domain.

	Domain	Search terms
1	Outcome: Fatigue^1,2^	(exhaustion) OR (exhausted) OR (fatigue MeSH) OR (tiredness) OR (burnout) OR (fatigue*) OR (weariness) OR (weary) OR (lethargy OR lethargic)
	AND	
2	Measurement method: Fatigue assessment^1,2^	(facit-f) OR (functional assessment of chronic illness therapy fatigue scale) OR (fatigue severity scale*) OR (fatigue assessment scale*) OR (fatigue assessment) OR (chalder fatigue scale) OR (chalder fatigue questionnaire) OR (chalder fatigue*)
	AND	
3	Target population^1,2^	(inflammatory bowel disease) OR (arthritis) OR (thyroid disease) OR (kidney disease) OR (liver disease) OR (multiple sclerosis) OR (fibromyalgia) OR (cancer) OR (stroke MeSH) OR (Parkinson disease) OR (cerebral palsy) OR (chronic disease*) OR (chronic illness*) OR (long term condition*) OR (chronic condition*) OR (chronic disease MeSH) OR (chronic disorder*) OR (anemia) OR (long covid) OR (chronic fatigue syndrome) OR (chronic obstructive pulmonary disease) OR (diabetes) OR (heart diseases MeSH)
	AND	
4	Physical activity^1,2^	(exercise MeSH) OR (running) OR (jogging) OR (swimming) OR (walking) OR (stair climbing) OR (physical activity) OR (physical activit*) OR (physical function) OR (physical fitness) OR (exercise therapy) OR (activit* of daily living) OR (therapeutic exercise) OR (rehabilitation) OR (aerobic activit*) OR (aerobic exercise) OR (strength training) OR (resistance training) OR (endurance activit*)

MeSH, Medical Subject headings.

* Used to identify all words beginning with the stem.

^1^Restricted search to title, abstract and keywords.

^2^Limit to English results.

### Eligibility criteria

Randomised controlled trials of PA interventions for adults with chronic diseases with fatigue reported as a primary or secondary outcome were included. Eligible RCTs included adult participants who were assigned randomly to a physical intervention or a control group. In addition, articles should have reported primary research studies in English. Excluded were protocol papers, editorials, discussion papers, and comments. Moreover, tailored interventions for the participants were excluded since this review aims to explore specific intervention programs that are based on objective and specific criteria for the PA intervention.

### Data management and screening

The EndNote software version 20.4 was used to remove the duplicates and the remaining results were imported to Rayyan^57^, which is a web tool designed for systematic reviews. Titles and abstracts were screened by two independent reviewers (IB and KES) to determine whether they met the eligibility criteria. The non-eligible abstracts were rejected, and numbers were documented. The full texts of potentially eligible studies were retrieved and assessed independently by IB and KES. All decisions of inclusion or exclusion were automatically recorded in Rayyan, and reviewers were blinded to each other’s decisions. Any disagreements were discussed and resolved by consensus between the two reviewers or by consulting a third reviewer (FJH). The outcome data that were used for the meta-analysis were extracted by IB. Uncertainties about outcome data were discussed with KES and USA and the original paper was accessed to reach an agreement.

### Risk of bias assessment

The risk of bias was assessed by two independent reviewers (IB, KES) using the Cochrane risk of bias tool consisting of five domains (version 2, ROB2)^58^. This approach addresses the following domains: the randomization process, the effect of assignment or adhering to intervention, missing outcome data, measurement of the outcome, and selection of the reported result. Each domain is scored as “low”, “some concerns”, or “high risk” of bias. Then, an overall risk of bias for each trial is provided through the tool’s algorithm. Disagreements about the risk of bias assessments were discussed and resolved by consensus by referring to the full text. ROB2 tool was used to create the risk of bias figures.

### Statistical analysis

The primary outcomes in the meta-analysis included the effect of different PA interventions on perceived fatigue as a first step. Then, the effects of the length and total sessions of interventions and the mode of PA on perceived fatigue were investigated as a second step in which we aimed to explore different ingredients of the interventions. In the analyses, the studies were distributed in different subgroups based on:

- the length of the interventions (2–6 weeks, 7–10 weeks, 11–15 weeks, 16 + weeks)
- the total sessions of the interventions (8–16 sessions, 18–24 sessions, 30–36 sessions, 45–48 sessions, 54 + sessions)
- the mode of PA (aerobic running, aerobic cycling, balance, resistance, combination, exergaming aerobic, horseback riding)

In addition, meta-analysis was conducted for the studies that included the post-trial follow ups, which assesses the effect retained at follow up, thus indicating the long-term effects. Random effects models were used for the inverse variance meta-analysis conducted in the Review Manager software (Review Manager 5.4; The Nordic Cochrane Centre, The Cochrane Collaboration). Because the interventions used different scales for the fatigue assessment, we performed standardised mean differences (SMD). SMD with 95% confidence intervals (CI) was used to describe the experimental and control group differences for post-intervention values in PA intervention subgroups as well as the overall effect. In addition, post-trial follow-ups were considered if appropriate. SMD values of 0.2, 0.5, and 0.8 represent a low effect, a moderate effect, and a large effect, respectively^59^. When the potentially eligible articles did not report mean (M) and standard deviation (SD), the corresponding authors were contacted by email to request the data. The articles were excluded from the meta-analysis if there was no reply within two weeks; however, they were included in the systematic review.

Statistical heterogeneity among studies was assessed by calculating the I^2^ index. Low heterogeneity was considered when I^2^ ≤ 25%, moderate when I^2^ ≤ 50% and > 25%, and high when ≤ 75% and > 50%^60^. Subgroup analysis was used to analyse the effectiveness of the PA interventions.

Moreover, among 31 studies, included in our analysis, five studies consisted of two experimental groups and one control group^61–65^. To ensure consistency, we examined whether the groups within each study belonged to the same PA mode. If both groups shared the same PA mode as well as intervention length, and total sessions, they were combined for the meta-analysis. Conversely, if different PA modes were used within the same study experimental groups, they were analysed separately.

### Publication bias

Publication bias was conducted in RStudio using Egger’s test on the meta-analysis data^66,67^, which evaluates funnel plot asymmetry. The level of statistical significance was set to α < 0.05.

## Results

### Search result

**Figure 1:**
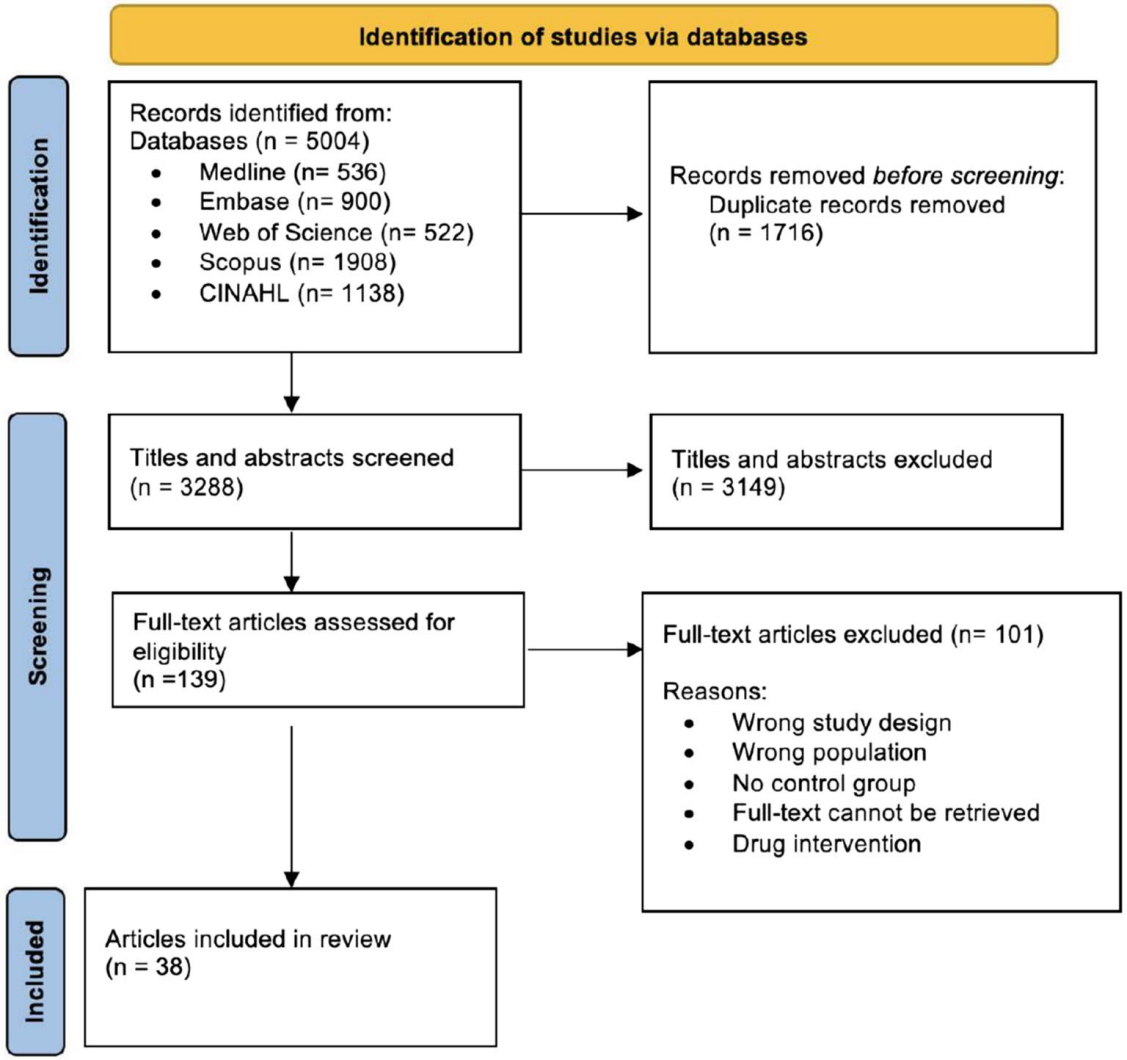
Flow diagram of trial selection, adapted from PRISMA.

Figure 1 demonstrates the search process in a flow diagram. A total of 5004 unique articles were identified from our initial search of five databases, while 3288 titles and abstracts were screened after removing the duplicates. Following, 139 potentially eligible full text articles were assessed. After the screening process, 38 studies were included in this systematic review.

### Characteristics of included studies

The included studies involved 2091 participants from different medical condition populations (receiving a PA intervention: n = 1071, controls: n = 1020). The subjects in all studies were adults, aged 19–89 years. The gender of subjects in those 38 studies was: female = 75.37% and male = 24.63%. All the studies were conducted in adults with the following chronic disorders: multiple sclerosis (n = 17)^61–64,68–79^, cancer (n = 11)^80–91^, chronic obstructive pulmonary disease (n = 1)^65^, fibromyalgia syndrome (n = 1)^92^, Parkinson's disease (n = 3)^37,93,94^, axial spondylarthritis (n = 1)^95^, bowel syndrome (n = 1)^96^, stroke (n = 1)^97^, rheumatoid arthritis (n = 1)^98^ and kidney failure (n = 1)^99^.

Table 2 presents the characteristics of the included studies and PA interventions. The characteristics include the sample size and subjects’ age and body mass index (BMI), the fatigue assessment, the mode, description, and intensity of PA, the duration and frequency of the PA interventions as well as its adherence.

**Table 2:** Characteristics of the included studies.

Lead author, (Year)	Chronic disorder/sample size	Participants' characteristics	Fatigue scale	Mode of PA	PA Description	Duration & frequency of PA	Length of intervention	Post-trial follow up	Control intervention	Adherence to intervention	Fatigue outcome
Ahmadi, et al. 2010	MS IG:11 CG:10	Age:32.27 ± 8.68 36.7 ± 9.32	FSS	Balance training	Hatha yoga	3 sessions/week, 60–70 min Stretching techniques, standing, supine, prone-lying, and sitting postures Pose holding time: 10–30 s, rest time: 30–60 s Intensity: not reported	8 weeks	No	Wait-list	Not reported	Significant improvement on FSS for IG (p = 0.01) (CG: p = 0.82)
Ahmadi, et al. 2013	MS IG:10 CG:10	Age: 36.8 ± 9.17 36.7 ± 9.32	FSS	Aerobic training	Treadmill running	3 sessions/week, 30 min treadmill + 10 min stretching Intensity: 40–75% age predicted maximal HR	8 weeks	No	Wait-list	Not reported	Fatigue levels were significantly lower for IG (p = 0.001)
Andreu-Caravaca, et al. 2022	MS EG:18 CG:12	Age: 44.89 ± 10.62 / 48.36 ± 10.23	FSS	Resistance training	Fast-velocity concentric resistance training	3 sessions/week, 5 min warm-up, bilateral leg press, unilateral leg extension, unilateral hip extension, and bilateral seated calf raise on conventional weight machines Intensity: 60–75% RM	10 weeks	No	Not reported	Not reported	Fatigue was improved in the EG (p = 0.004)
Cakıt, et al. 2010	MS EG (1):14 EG (2):10 CG:9	Age: 36.4 ± 10.5 43.0 ± 10.2 35.5 ± 10.9 BMI (kg/m^2^): 25.2 ± 4.7 24.6 ± 5.2 21.3 ± 1.6	FSS	(1) Resistance training (2) Combination training	(1) Cycling progressive resistance training (2) Home exercise program for strength & balance	(1) 2 sessions/week (static bicycle ergometer) 15 sets/reps, 2 min high resistance pedalling * 2 min low-resistance or 2 min rest 5 min warp-up activities & stretches, 20–25 min balance exercises, 5 min whole-body stretching Intensity: High resistance (2) 2 sessions/week 5 min warp-up activities & stretches, 20–25 min balance exercises, 5 min whole-body stretching Intensity: Not reported	8 weeks	No	Continue with their normal living	(1) 93% (2) 60%	(1) Significant changes between EG and CG (p < 0.05) (2) non-significant difference between EG and CG (3) Significant difference between two EG in favour of EG 1 (p < 0.05)
Cerulli, et al. 2014	BC IG:10 CG:10	Age: 45.3 ± 4.32 46.0 ± 2.78 BMI (kg/m^2^):23.51 ± 2.89 25.81 ± 4.82	FACIT-F	Balance training	Horseback riding	2 sessions/week, 60 min therapeutic riding 3 phases of riding treatments: (1) warp-up, horse caring, (2) riding, (3) unsaddling Intensity: Not reported	16 weeks	No	Continue with their normal living	Not reported	FACIT-F scores for IG (p = 0.004)
Castro-Sanchez, et al. 2012	MS EG: 36 CG: 37	Age: 46 ± 9.97/ 50 ± 12.31	FSS	Strengthen training	Aquatic exercise Ai Chi	2 sessions/week, 60 min Intensity: Not reported	20 weeks	No	Relaxation	Not reported	Significant reduction in fatigue at weeks 20 (p < 0.043) for EG No significant differences for CG
Deijle, et al. 2022	Stroke EG:60 CG:59	Age: 64.7 ± 8.9 / 63.9 ± 10.6	FSS	Combination training	Aerobic and strength training	2 sessions/week, 60 min Aerobic: cycle ergometer, treadmill, or rowing machine Intensity 40–80% THR Strength: weight machines, 3 sets, 10–12 reps Intensity 60–70%	12 weeks	24 months	Usual care	100%	Significance was found between‐group difference for fatigue, in favour of the EG at 12 months (mean difference, 0.6 out of 63)
De Luca, et al. 2016	BC IG:10 CG:10	Age: 50.2 ± 9.7 46.0 ± 2.8 BMI (kg/m^2^): 24.6 ± 3.6 25.81 ± 4.8	FACIT-F	Combination training	Aerobic and strength training	2 sessions/week, 90 min 10 min warm-up and cool-down period (cycle ergometer pedalling), resistance training (weightlifting), aerobic (stationary bike) Intensity: Gradually increased	24 weeks	No	Continue with their normal living	Not reported	FACIT-F scores for IG (p = 0.017) FACIT-F scores for CG (p = 0.484)
D’Silva, et al. 2022	IBS IG:38 CG:41	Age: 43.50 ± 12.9 / 47.10 ± 14.8	MFIS	Balance training	Upa Yoga	1 session/week, 60 min	8 weeks	No	Received 10 min education on IBS	79%	Significant difference was found on fatigue between two groups (p = 0.035)
Duruturk, et al. 2015	COPD EG (1):15 EG (2):14 CG:13	Age: 61.2 ± 5.0 61.2 ± 5.1 63.8 ± 5.7 BMI (kg/m^2^): 26.7 ± 3.9 28.4 ± 5.3 25.4 ± 3.5	FSS	(1) Aerobic (2) Combination training	(1) Cycling (2) Calisthenic	(1) 3 sessions/week, 20–30 min Warm-up/Cool-down: 3 min pedalling low intensity Intensity: High intensity 50–70% VO2max (2) 3 sessions/week, 20–45 min 16 different rhythmical, calisthenic exercises (strengthening & stretching) Intensity: Moderate intensity (Borg Scale)	6 weeks	No	Received education session and continued their current medical therapies	(1) 94% (2) 89	1) Significantly improved between EG and CG (p < 0.001) 2) Significantly improved between EG and CG (p < 0.001) 3) Non-significant between EG (p = 0.31)
Escudero-Uribe, et al. 2017	MS EG (1):14 EG (2):16 CG:18	Age: 40.3 ± 8.9 43.1 ± 10.2 43.0 ± 9.3 BMI (kg/m^2^): 23.5 ± 2.9 24.2 ± 3.5 27.2 ± 6.9	FSS	(1) Combination training (2) Combination training	(1) Balance trainer (2) Whole-body vibration	(1) 2 times/week, 60–100 min (Balance Trainer used) 5 min warm-up/cool-down, 15–30 min aerobic (e.g., bike, walking, elliptical) 15–30 min circuit exercises (body weight, coordination, balance) (Balance trainer used) 15 min strength exercises (major muscle groups) Intensity: Light—somewhat hard (Borg Scale) (2) 2 sessions/week, 60–100 min 5 min warm-up/cool-down, 15–30 min aerobic (e.g., bike, walking, elliptical) 15–30 min circuit exercises (body weight, coordination, balance) (Whole-body vibration used) 15 min strength exercises (major muscle groups) Intensity: Light—somewhat hard (Borg Scale)	12 weeks	No	Wait-list	(1) 79.5% (2) 80%	(1) Significant improvement on EG (p < 0.05) (2) Significant improvement on EG (p < 0.05)
Etnier, et al. 2009	FMS EG:8 CG:8	Age: 54.69 ± 9.25 (All participants: N = 16)	FSS	Combination training	Walking & circuit training program	3 sessions/ week, 60 min Walking, resistance exercises, stretching Intensity: 55%-65% MHR	18 weeks	No	Wait-list	65%	Significant difference in EG (p < 0.01)
Headley et al. 2004	BC IG: 16 CG: 16	Age: 52.25 ± 11.43/50.0 ± 7.10	FACIT-F	Balance training	Dynamic, balance exercises	3 sessions/week, 30 min seated exercise program, moderate-intensity repetitive motion exercises Intensity: Moderate	13 weeks (4 cycles of chemotherapy)	No	Continue with their usual activities or exercises	75%	FACIT–F scores for the entire sample declined at a significant rate (p = 0.003) beginning with cycle 3 but at a slower rate for the experimental group (p = 0.02)
Furtado de Oliveira, et al. 2018	C IG:19 CG:19	Age:61.46 ± 8.79 57.62 ± 7.57 BMI (kg/m^2^): 28.36 ± 4.94 28.06 ± 3.74	FACIT-F	Aerobic exercise	Exergaming Xbox360/Kinect	2 or 3 sessions/week, average 2 min per game*9 times Xbox360/Kinect Game: Your Shape Fitness Evolved, Subgames: “Stomp It”, "Wall Breaker" Intensity: mild to moderate intensity:	8–10 weeks	No	Not reported	Not reported	IG had lower values than CG (p = 0.007)
Gomez-Illan, et al. 2020	MS EG:13 CG:13	Age: 45.31 ± 11.06 41.31 ± 9.58	FSS	Strength Training	Strength training exercises	3 sessions/week 5 min cardiovascular exercises + strength training (e.g., biceps/leg curls, pulldown, leg extension, calf raises, leg press) Cool-down & stretching Intensity: Moderate & vigorous	8 weeks	10 weeks follow up	Continue with their normal living	Not reported	Significantly reduced of fatigue scores in EG (p < 0.001)
Kim, et al. 2019	CC EG:37 CG:34	Age: 55.7 ± 8.7 56.8 ± 10.2 BMI (kg/m^2^): 23.7 ± 2.9 23.3 ± 3.6	FACIT-F	Combination training	Aerobic & resistance training	Home-based exercise programme Aerobic > 10,000 steps every day Strength training: 30 min 1st 6 weeks, moderate: 3 sets* 7 core & resistance exercise (12–15 reps/set) 2nd 6 weeks, vigorous: 3 sets* 5 aerobic & resistance combined exercises (12–15 reps/set) Intensity: Moderate & vigorous	12 weeks	No	Continue with their usual activities or exercises	Not reported	No significant change
Koevoets, et al. 2022	BC IG:91 CG:90	Age: 52.1 ± 8.6 / 52.5 ± 8.7	MFI	Combination training	Aerobic & strength training	4h/week Strength: 2h/week Intensity: increased gradually Aerobic: 2h/week, Nordic/power walking Intensity 55–65% HRR	26 weeks	No	Requested to maintain their habitual physical activity level	≥ 80%	Significant beneficial intervention effects were found for fatigue (effect size 0.56)
Langeskov-Christense, et al. 2021	MS IG:43 CG:43	Age:44.0 ± 9.5 45.6 ± 9.3 BMI (kg/m^2^):25.5 ± 4.6 24.3 ± 3.7	FSS	Aerobic training	Cycling	2 sessions/week, 30–60 min (1 continuous/1 interval) Cycling, rowing, cross training Intensity: 65–95% MHR	24 weeks	24 weeks follow up	Wait-list	93.30%	No changes observed in FSS
Loeppenthin, et al. 2022	RA IG:17 CG:21	Age: 57.8 ± 9.8/ 54.8 ± 9.6	Overall fatigue (VAS)	Aerobic exercise	Bicycle ergometers	3 sessions/week, 20–30 min Intensity: 40–80%	6 weeks	No	Usual care and maintained normal activities	High rates	Fatigue was significantly lower in the IG than in the CG. Between-group differences were overall fatigue (p = 0.001)
Mostafaei, et al. 2021	BC IG:30 CG:30	Age: 48.46 ± 5.72/49.60 ± 7.48	FSS	Combination training	Dynamic, balance exercises	3 session/week, 30 min 5 min warm-up/ cool-down, 20 min stretching, dynamic, resistant band, & balance exercises Intensity: Not reported	6 weeks	1 month follow up	No exercise	Not reported	Significant difference was observed between IG & CG (p = 0.001)
Ozkul, et al. 2018	MS EG:18 CG:18	Age: 35.83 ± 9.45 36.5 ± 8.83 BMI (kg/m^2^): 23.79 ± 4.23 25.28 ± 3.89	FSS	Combination training	Aerobic & Pilates training	3 sessions/week, 60 min Aerobic: 5 min warm-up/cool-down, 20 min walking (treadmill) 15 min rest Pilates: warm-up, exercise balls, elastic bands exercises (10–20 reps), stretching, posture & relaxation exercises Intensity: 60–80% MHR	8 weeks	No	Home-bases: Relaxation exercises (3 sessions/week)	85%	Significantly improved in EG (p < 0.05)
Ozkul, et al. 2020	MS EG (1):13 EG (2):13 CG:13	Age: 35.54 ± 11.33 32.31 ± 10.08 35.54 ± 8.25 BMI (kg/m^2^): 25.53 ± 3.78 22.94 ± 4.05 23.53 ± 3.35	FSS	(1) Balance training (2) Balance training	(1) Pilates & balance (2) Virtual Reality-RAGU system	1) 2 sessions/week, 60 min Pilates: 30 min, 10 min rest (warm-up/cool-down) Balance training exercises: 20 min (10–20 reps) (e.g., hitting/avoiding the ball) Intensity: Not reported 2) RAGU system Pilates: 30 min, 10 min rest (warm-up/cool-down) 20 min of 2 games for balance (football, guillotine), 5 min between games Intensity: Not reported	8 weeks	No	Progressive relaxation exercises (2 sessions/week)	(1) 82.7% (2) 80.77	(1) Significantly improve in EG (p = 0.006) (2) Significantly improve in EG (p = 0.001)
Parent-Roberge et al. 2020*	C EG: 10 CG: 10	Age: median 67.5 (67.0–69.8) 69.5 (68.2–73.5)	FACIT-F	Combination training	Aerobic & resistance training	3 sessions/week Aerobic exercise training 20–40 min Resistance training: multi-joint exercises targeting lower limb, upper limb and one core exercise (1–3 sets, 12–15 reps) Intensity: 40–75%MHR	12 weeks	No	static stretching 2 sessions/week	No reported	Non-significant (p = 0.50)
Parvan, et al. 2017	KF EG:23 CG:23	Age: 89.04 ± 9.51 60.17 ± 10.52	FSS	Aerobic training	Cycling	3 sessions/week, 30–60 min Pedal bicycle (stationary bike) Intensity: 0–60 RPM	8 weeks	No	Not reported	Not reported	Significantly reduced in EG (p < 0.05)
Pan et al. 2022	MS EG (1): 30 EG (2): 30 CG: 20	Age: 42.23 ± 5.14 / 40.93 ± 4.76 / 42.25 ± 4.52 BMI: 22.98 ± 2.17 / 23.45 ± 2.88 / 23.56 ± 3.15	FSS	Balance training	1)Baduanjin exercise 2)Yoga	1) 60 min/day (8 movements for limbs, body-trunk and eye movements) Intensity: Not reported 2) 60 min/day (yoga, respiratory exercises) Intensity: Not reported	24 weeks	No	CG was advised not to seek any other regular exercise	Not reported	Baduanjin and yoga were both effective in improving balance ability, and alleviating fatigue
Petajan, et al. 1996	MS EG:21 CG:25	Age: 41.1 ± 2.0 39.0 ± 1.7	FSS	Aerobic training	Cycling	3 sessions/week, 50 min Cycle ergometer: 5 min warm-up/cool-down, 30 min aerobic 5–10 min stretching Intensity: 73% MHR	15 weeks	No	Continue with their usual activities or exercises	97%	Significantly reduced at 10 weeks (p < 0.05) No significant changes at 15 weeks
Ribas, et al. 2017	PD EG:10 CG:10	Age: 61.7 ± 6.83 60.20 ± 11.29 BMI (kg/m^2^): 24.85 ± 3.08 25.01 ± 2.73	FSS	Combination training	Exergaming Wii Fit	2 session/week, 30 min 7 Wii Fit games (Nintendo): Table Tilt, Tilt City, Penguin Slide, Soccer Heading, Basic Run, Obstacle Course & Basic Step 30 Intensity: Not reported	12 weeks	2 months follow up	Not reported	Not reported	Significant difference in EG (p = 0.000)
Rios Romenets, et al. 2015	PD EG:18 CG:15	Age: 63.2 ± 9.9 64.3 ± 8.1	FSS	Balance training	Dance	2 sessions/week, 60 min Traditional Argentine Tango Intensity: Not reported	12 weeks	No	Wait-list	Not reported	Non-significant (EG: p = 0.167, CG: p = 0.138) Between EG & CG borderline significant (p = 0.057)
Surakka et al. 2004	MS EG: 49 CG: 50	Age: 44 ± 6 / 44 ± 7	FSS	Combination training	Aerobic & resistance training	Aerobic program: 5–7 min warming up, 20–25 min aerobic exercises, 5–8 min cooling down Resistance program: 10 exercises with 10–15 repetitions/ 2 sets Intensity: 65–70%	23 weeks	3 weeks follow up	CG continued with their normal living	Not reported	No significant group interactions were observed in fatigue
Steindorf, et al. 2014	BC EG:77 RC: 78	Age: 55.2 ± 9.5 56.4 ± 8.7 BMI (kg/m^2^):26.9 ± 5.4 27.6 ± 4.8	FAQ	Resistance training	Strength training exercises	2 sessions/week, 60 min 8 machine-based resistance exercises Intensity: 60–80% of 1 RM	12 weeks	No	Muscle-relaxation program	97%	Significant between-group mean differences favouring EG were observed for general fatigue (p = 0.044)
Straudi, et al. 2014	MS EG:12 CG: 12	Age: 49.92 ± 7.51 55.25 ± 13.82	FSS	Combination training	TOCT	5 sessions/week, 120 min 7 workstation: step, slalom, tandem exercise, goals, obstacles, long step, Treadmill (30 min walking) Intensity: High	2 weeks	3 months follow up	Continue with their normal living	Not reported	No significant change
Sveaas, et al. 2020	AS EG:50 CG: 50	Age: 45.1 ± 10.7 47.2 ± 10.3 BMI (kg/m^2^): 27.4 ± 5.0 28.6 ± 6.3	FSS	Combination training	Cardiorespiratory & strength	3 sessions/week, 60 min Treadmill or cycle ergometer: 10 min warm-up, (4 min walking/running/cycling, 3min rest) *4 20 min squats, leg press, pulldowns, and sit-ups (2–3 sets, 8–10 reps) Intensity: 90–95% MHR	12 weeks	9 months follow up	Continue usual physical activity	≥ 80%	Significant beneficial effect between EG & CG (p = 0.01)
Tarakci, et al. 2013	MS EG:51 CG: 48	Age: 41.49 ± 9.37 39.65 ± 11.18	FSS	Combination training	balance coordination & functional exercises	3 sessions/week, 60 min Flexibility, range of motion, strengthening with/without bands (lower stability, core stabilization, balance, coordination) Intensity: Moderate	12 weeks	No	Wait-list	Not reported	Significant change in EG (p = < 0.001) Significant negative effect in CG (p = 0.002) Non-significant between EG & CG (p = 0.733)
Van Den Berg, et al. 2006	MS EG:8 CG: 8	Not reported	FSS	Aerobic training	Treadmill running	3 sessions/week, 30 min Treadmill Intensity: 55–85% APMHR	4 weeks	No	Wait-list	Not reported	No significant change
Wei, et al. 2022	BC IG: 35 CG:35	Age: median 52 / 55 BMI: 22.86 ± 2.55 / 23.26 ± 2.56	MFSI	Balance training	Baduanjin exercise	5 sessions/week, 30 min, exercises consists of 10 postures Intensity: low-moderate	12 weeks	No	Maintain their usual healthy lifestyle	Not reported	No significant change in fatigue (p = 0.610)
Winward, et al. 2011	PD IG:20 CG:17	Age: 63.4 ± 6.7 64.9 ± 9.6	FSS	Combination training	Aerobic & strength training	5*30 min aerobic & 2 strength sessions/week, 30–45 min Cardio, muscle strength & flexibility Community gym-based exercise program Intensity: Not reported	12 weeks	No	Wait-list	Mean 15 sessions 55% 1 or more session per week for 12 weeks	No significant change in fatigue (p = 0.76)
Yazgan, et al. 2020	MS EG (1):12 EG (2):15 CG:15	Age: 43.08 ± 8.74 47.46 ± 10.53 40.66 ± 8.82 BMI (kg/m^2^): 24.05 ± 4.21 25.52 ± 4.47 23.91 ± 4.86	FSS	(1) Balance training (2) Balance training	(1) Balance trainer (2) Nintendo Wii Fit	(1) 2 sessions/week, 60 min (Balance trainer used) 10 min warm-up/cool-down Different games (Collect apples, Outline, Paddle War, Evaluation of Movement games) Intensity: Not reported (2) 2 sessions/week, 60 min 10 min warm-up/cool-down Different games (Penguin slide, Table Tilt, Ski Slalom, Balance Bubble) Intensity: Not reported	8 weeks	No	Wait-list	Excellent	(1) Significant change in EG (p = 0.002) (2) Significant change in EG (p = 0.002) (3) Significant change in favour of the 2 EG compared to CG (p < 0.001)
Yee, et al. 2019	BC EG:8 CG:6	Age: 60.1 ± 12.7 65.0 ± 6.9 BMI (kg/m^2^): 28.4 ± 6.2 28.1 ± 5.6	FACIT-F	Resistance training	Strength training exercises	Home-based exercise programme, 40–55 min 16 total exercise sessions, 2 sets/ 10–12 reps, 1 min recovery brist walk 10–15 min, 30–40 min resistance training (chest press, row, bicep curl, calf raises, lunges, squats) Intensity: Moderate	8 weeks	8 weeks follow up	Continue with their normal living	walking 25%, resistance 100%	Reduction of fatigue in the EG (ES = 0.93)

PA, physical activity; MS, multiple sclerosis; BC, breast cancer; COPD, chronic obstructive pulmonary disease; FMS, fibromyalgia syndrome; CC, colorectal cancer; C, cancer; KF, kidney failure; PD, Parkinson's disease; BS, irritable bowel syndrome; RA, rheumatoid Arthritis; AS, axial spondylarthritis; FSS; fatigue severity scale; FACIT-F, functional assessment of chronic illness therapy—fatigue; MFIS, modified fatigue impact scale; MFI, multidimensional fatigue inventory; VAS, visual analogue scale; FAQ, fatigue assessment questionnaire; MFSI, multidimensional fatigue symptom inventory; IG, intervention group; EG, exercise group; RC relaxation group; CG, control group; RM, repetition maximum; MHR, maximum heart rate; THR, target heart rate; HRR, heart rate reserve; RPM, revolutions per minute; MREP, repetition maximum for weight training; mins, minutes; MET, metabolic equivalent of task; ES, effect size; TOCT, task-oriented circuit training; RAGU, Augmented Reality Applications in Rehabilitation*not included in the meta-analysis due to missing values of M(SD).

The fatigue severity scale (FSS) was used in twenty-three studies and was the most frequently used fatigue measure among the thirty-eight studies. The FSS is a validated and reliable 9-item questionnaire designed to assess the impact of perceived fatigue among adults diagnosed with chronic conditions^100–102^. The functional assessment of chronic illness therapy—fatigue scale (FACIT-F) was used in seven studies. FACIT-F (version 4) is a 40-item questionnaire evaluating self-reported fatigue and its influence on everyday activities and function among adults with cancer and older people who experience fatigue^103^. It is valid and reliable in several patients’ populations^104–106^. One study used the fatigue assessment questionnaire (FAQ), which is a validated 20-item questionnaire assessing fatigue among adults with cancer^107,108^. The modified fatigue impact scale (MFIS) was used by one study and contains 9 items^109^. MFIS provides an assessment of the effects of fatigue in physical and cognitive functioning and is reliable and valid in several clinical populations^110^. The multidimensional fatigue inventory (MFI) is 20-item questionnaire evaluating fatigue and was used by one study^111^. MFI is valid and reliable in several chronic conditions^112,113^. The visual analogue scale (VAS) is an 18-item questionnaire evaluating fatigue and is valid and reliable in stroke population^114^. The multidimensional fatigue symptom inventory (MFSI) is a 30-item questionnaire and was used by one study. It measures fatigue and it is valid and reliable in cancer populations^115^. Among the 38 PA interventions, eight used aerobic training, thirteen interventions used balance training, while sixteen interventions used a combination training of strength, balance, and/or aerobic. Strength training was used by six PA interventions. Two studies used dance (Tango), and horse-riding training.

### Risk of bias

**Figure 2:**
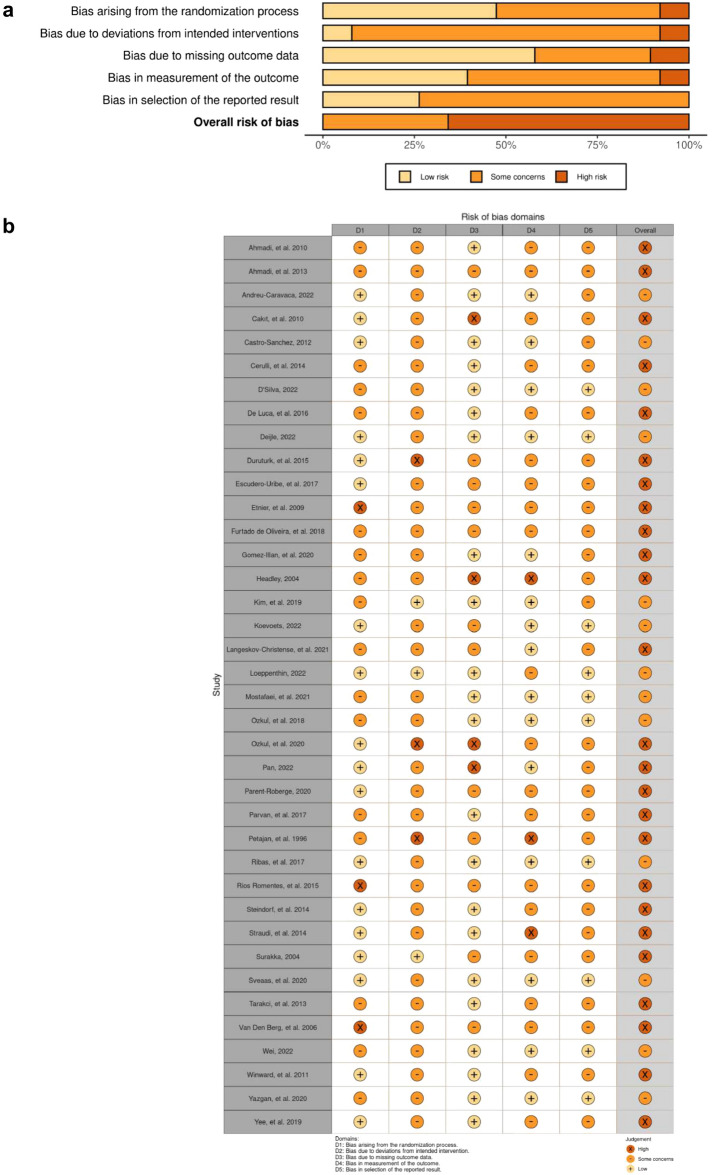
(**a**) Risk of bias graph. (**b**) Risk of bias summary.

The Cochrane tool was used to assess the risk of bias in included studies (n = 38). Figure 2a and b show the risk of bias analysis. Overall, twenty-five studies showed a high risk of bias, and thirteen studies showed some concerns. The trials by Etnier et al.^92^, Rios Romenets et al.^94^ and Van Den Berg et al.^75^ presented a high risk of bias in the domain of randomization process. Whereas 18 trials showed a low risk of bias in this domain^37,62–65,74,77,79,84,85,87,89,90,93,95,97,98,116^. The trials conducted by Duruturk et al.^65^, Ozkul et al.^72^ and Petajan et al.^73^ showed high risk of bias in the deviations from intended intervention. While only three trials presented as low risk^77,83,98^. In the domain of missing outcome data, 22 trials presented a low risk of bias^37,61,68,70,72,74,76,79–81,83–86,90,91,93,95–99^ while only four presented a high risk of bias^62,64,78,88^. 15 trials showed a low risk of bias^61,70–72,78,79,83,86,87,90,91,93,95–97^ in the measurement of outcome domain while three showed a high risk of bias^73,74,88^. Ten trials presented a low risk of bias^61,72,86,87,91,93,95–97,117^ in the selection of reported results while no trials presented a high risk.

### Effects of PA interventions on perceived fatigue

**Figure 3:**
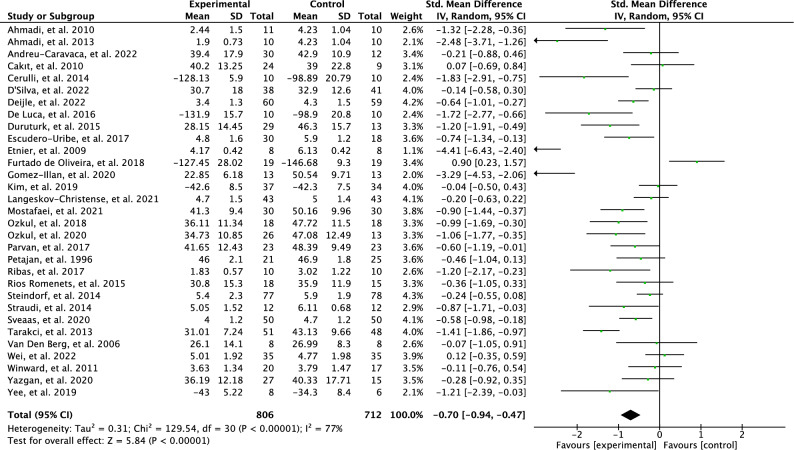
Forest plot of effects of physical activity interventions on perceived fatigue.

Initially, a meta-analysis was conducted to investigate the effects of all the included PA interventions on perceived fatigue. Eventually, 31 articles out of the 38 were included in the meta-analysis. The test for overall effect indicates that there is a moderate effect for reduction in perceived fatigue based on random effects model (SMD = 0.70; 95% CI = 0.94 to 0.47; *p* < 0.00001) with high heterogeneity results between studies (I^2^ = 77%). The outcomes are illustrated in Fig. 3.

**Figure 4:**
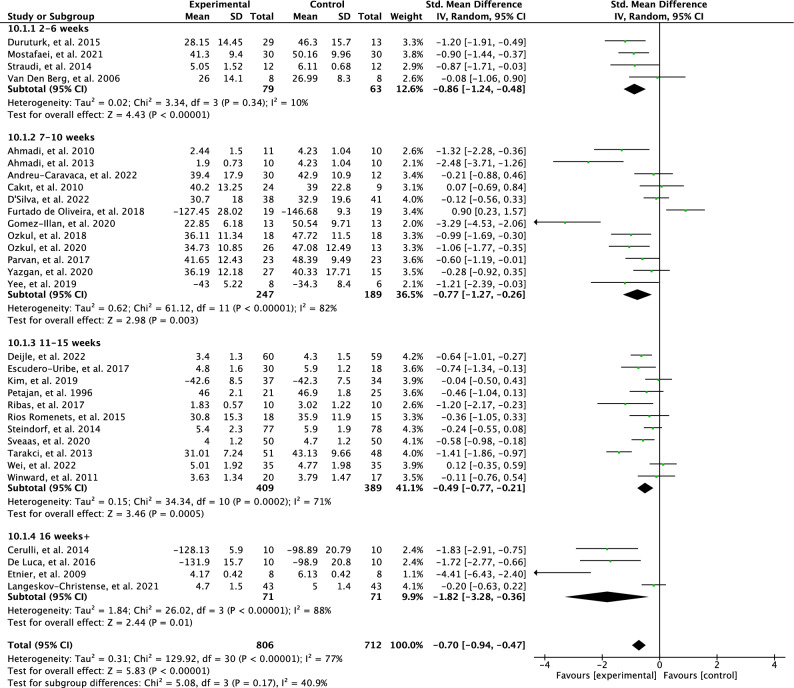
Forest plot of effects of physical activity interventions length on perceived fatigue.

The effects of the intervention length on perceived fatigue are presented in Fig. 4. The test for subgroup differences was not statistically significant with moderate heterogeneity (p = 0.17, I^2^ = 40.9%). Interventions that lasted for 2–6 weeks have a larger effect (SMD = 0.86; 95% CI = 1.24 to 0.48; *p* < 0.00001) compared to interventions that were 7–10 weeks (SMD = 0.77; 95% CI = 1.27 to 0.26; *p* = 0.003). The results from the interventions that were 2–6 weeks showed low heterogeneity (I^2^ = 10%) while the results from the interventions that were 7–10 weeks showed high heterogeneity (I^2^ = 82%). Moreover, the interventions that were 11–15 weeks showed a low effect for perceived fatigue (SMD = 0.49; 95% CI = 0.77–0.21; *p* = 0.0005) and results were heterogeneous between studies (I^2^ = 71%). A high effect was found for the interventions that were 16 weeks + (SMD =1.82; 95% CI = -3.28 to -0.36; p = 0.01) and results were heterogeneous between studies (I^2^ = 88%).

**Figure 5:**
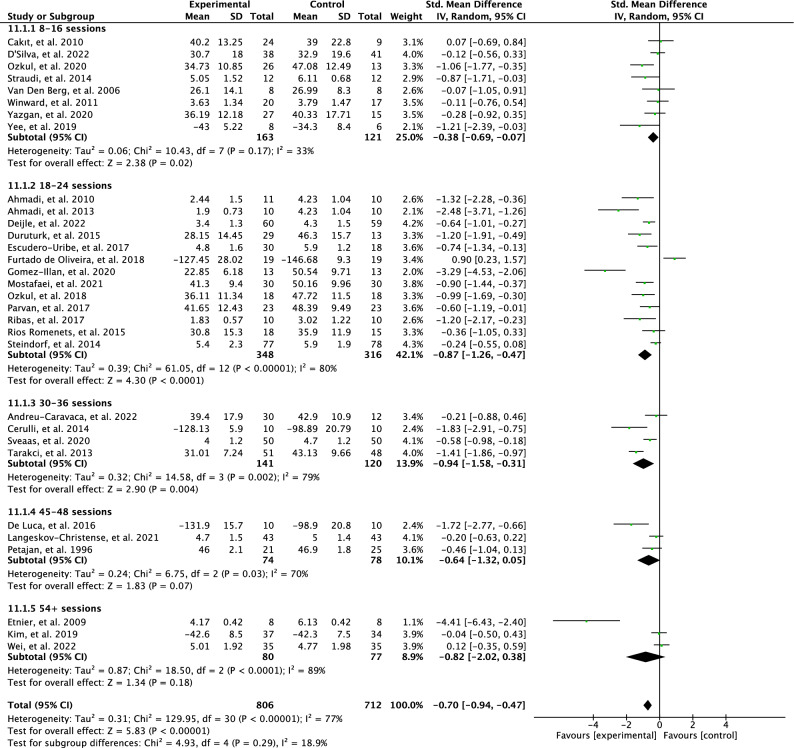
Forest plot of effects of total sessions of physical activity interventions on perceived fatigue.

The effects of the intervention total sessions of PA interventions on perceived fatigue are illustrated in Fig. 5. The test for the subgroup differences was not statistically significant with low heterogeneity (p = 0.29; I^2^ = 18.9%). Meta-analysis showed a low effect on perceived fatigue in the 8–16 sessions (SMD = 0.38; 95% CI = -0.69–0.07; *p* = 0.2) and results were moderately heterogeneous between studies (I^2^ = 33%). Interventions with 18–24 sessions had a large effect for perceived fatigue (SMD = 0.87; 95% CI = − 1.26 to − 0.47; *p* < 0.00001) and results were found heterogenous (I^2^ = 80%). The interventions with 30–36 sessions showed a high effect (SMD = 0.94; 95% CI = -1.58 to–0.31; *p* = 0.004) and heterogeneity was found between studies (I^2^ = 79%). Inversely, meta-analysis showed no effect for perceived fatigue in the interventions of 45–48 sessions (SMD = 0.64; 95% CI = -1.32 to– 0.05; p = 0.07) and results between the studies were heterogeneous (I^2^ = 70%). Similarly, interventions with 54+ sessions were found to have no statistically significant results (SMD = 0.80; 95% CI = − 2.02 to 0.38; p = 0.18, I^2^ = 89%).

**Figure 6:**
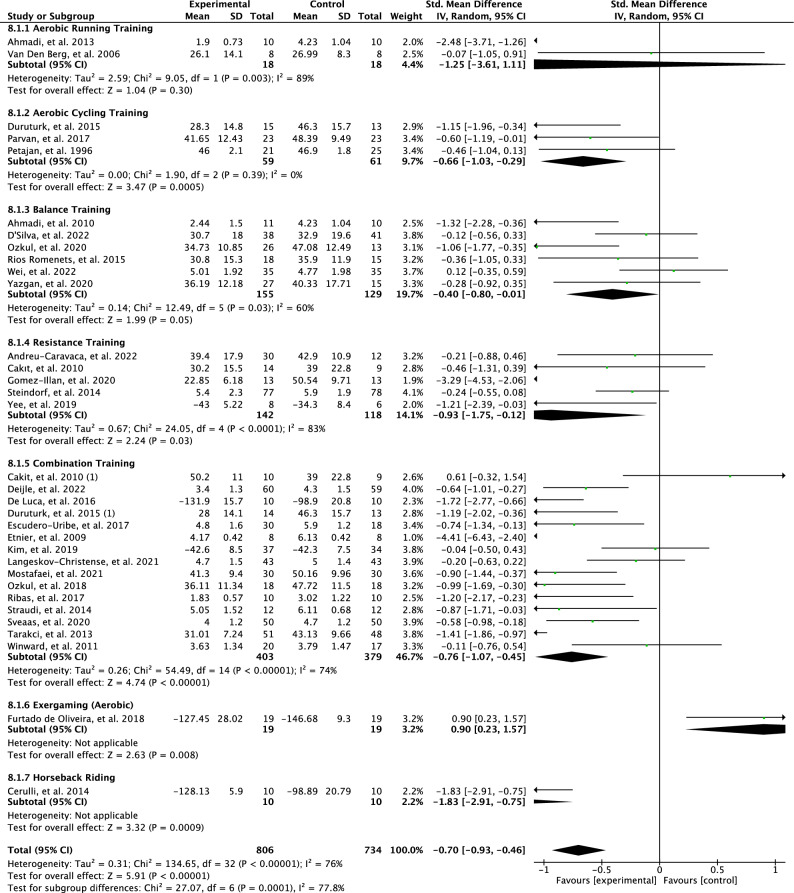
Forest plot of effects of modes of physical activity interventions on perceived fatigue.

The effects of the mode of PA interventions on perceived fatigue are illustrated in Fig. 6. Overall, the test for subgroup differences suggests that there is a statistically significant subgroup effect based on a random model with high heterogeneity (*p* = 0.001; I^2^ = 77.8%). Meta-analysis showed no effect for perceived fatigue in the aerobic running training subgroup (SMD = 1.25; 95% CI = − 3.61 to 1.11; *p* = 0.30) and results between studies were heterogenous (I^2^ = 89%). The estimates showed a moderate effect for perceived fatigue in the aerobic cycling training interventions (SMD = − 0.66; 95% CI = − 1.03 to − 0.29; *p* = 0.0005) and the results between studies were not heterogeneous (I^2^ = 0%). Interventions with balance training showed a low effect for perceived fatigue (SMD = − 0.40; 95% CI = − 0.80 to − 0.01; *p* < 0.05) and results between studies were moderately heterogeneous (I^2^ = 60%). Interventions with resistance training sshowed a high effect for perceived fatigue (SMD = 0.93; 95% CI = -1.75 to–0.12; *p* = 0.03, I2 = 83%). Interventions with combination training showed a moderate effect for perceived fatigue (SMD = 0.76; 95% CI = -1.07 to –0.45; p <0.00001) and results were heterogeneous between studies (I^2^ =74%). Interventions with aerobic exergaming and horseback riding were not included in the subgroup meta-analysis since there was only one study in each group.

**Figure 7:**
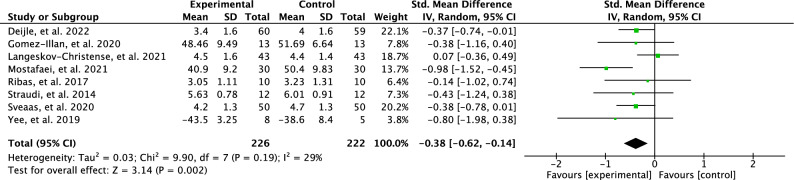
Forest plot of effects of post physical activity interventions follow up.

A meta-analysis was conducted for the post-trial follow ups. There were only eight studies that included post-trial follow up measurements. The test for the long-term effects showed a low effect for perceived fatigue (SMD = 0.38; 95% CI = -0.62 to–0.14; *p* = 0.002) and results were moderately heterogenous between studies (I^2^ = 29%). The outcomes are illustrated in Fig. 7.

### Publication bias

**Figure 8:**
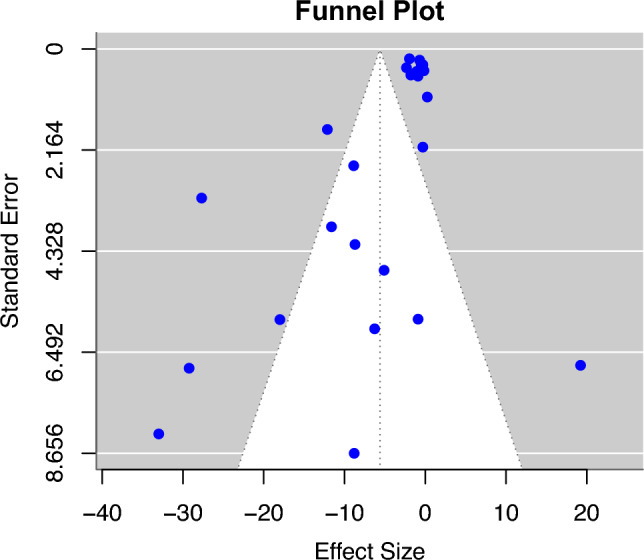
Funnel plot of publication bias on the meta-analysis data.

The publication bias assessment was estimated on the meta-analysis data. The analysis showed statistically significant evidence of publication bias (z = − 2.77, p=0.006). The funnel plot demonstrated visible asymmetry, indicating the possibility of publication bias. The estimated intercept of the regression line was -1.38 (95% CI: -5.45 to 2.68) when the standard error of the effect sizes approached zero. The funnel plot is illustrated in Fig. 8.

## Discussion

In this meta-analysis, data from 33 randomised control trials were synthesized to examine the effectiveness of PA interventions in reducing perceived fatigue among adults with chronic conditions. To the best of our knowledge, this is the first study to comprehensively evaluate the impact of PA interventions on perceived fatigue across a range of chronic conditions.

Firstly, this meta-analysis demonstrates that PA interventions have a moderate effect on perceived fatigue (SMD = 0.70) among adults with chronic conditions. Previous studies investigating the effects of PA on fatigue have yielded inconsistent findings. Some studies have demonstrated a positive association between exercise and fatigue in adults with chronic conditions^118,119^. Others, have reported that PA can reduce both fatigue and pain^33,35,120^, while contradictory results have indicated no effect of exercise on fatigue^37,121^. Our meta-analysis identifies a moderate effect of PA on fatigue reduction, providing new insights for the future design of targeted PA interventions to alleviate fatigue in individuals with various chronic conditions^121,124^.

Regarding the intervention length, this meta-analysis revealed that trials lasting from two to six weeks and sixteen to twenty-four weeks showed large effects on reducing perceived fatigue among adults with chronic conditions (SMD=0.86, 1.82, respectively). Low heterogeneity was found in two to six weeks intervention length, although the limited number of trials in this subgroup may be a limitation. High heterogeneity was found in the sixteen to twenty-four weeks subgroup, while limited trials in this subgroup could pose potential limitations. Additionally, adherence to the intervention was reported in two out of the four trials in the sixteen to twenty-four weeks subgroup^71,80,81,92^, with adherence rates of 65%^92^ and 93.3%^71^. The short-term interventions may benefit from immediate results as it can be something new to the participants, leading to a rapid reduction in the perceived fatigue. While longer interventions may result in more sustained changes over time, hence the larger effect in perceived fatigue. In literature, longer interventions (6-12 months) have been found to be effective in achieving behavior change for example in nutrition and PA^122,123^. However, in this study due to the limited number of trials in these subgroups, we cannot draw any conclusions. Additionally, seven to ten weeks demonstrated a moderate effect on perceived fatigue among adults with chronic conditions (SMD=0.77). The subgroup of eleven to fifteen weeks showed a moderate effect (SMD=0.49). However, high heterogeneity was observed in these two subgroups, potentially influenced by other intervention characteristics such as total sessions or mode of PA or even the disease diagnosis. The duration of an intervention holds importance for health professionals and researchers, as clinical and health interventions often face budget constraints^124^. While this meta-analysis suggests a trend of shorter PA interventions (up to 24 weeks) being effective for reducing fatigue, other factors may influence the results. Therefore, future research is necessary to determine the optimal and effective intervention duration, considering factors such as cost-effectiveness and time-efficiency in research and rehabilitation settings.

The exploration of total sessions in PA interventions yielded interesting findings. It was observed that interventions comprising 18–24 and 30–36 sessions had a substantial impact on perceived fatigue (SMD = 0.87 and 0.94, respectively). The considerable heterogeneity observed in the results could be partially attributed to other intervention characteristics, such as intervention duration and mode. Moreover, interventions comprising 8-16 weeks have a low impact on perceived fatigue (SMD=0.38). On the other hand, subgroups with 45–48, and 54+ total sessions showed no effect on perceived fatigue. The uneven distribution of studies among these subgroups might have limited the analysis of their effects on the total sessions of the interventions. Thus, researchers should be careful when interpreting the pooled effect sizes and focus on the observed data patterns. Furthermore, the duration of PA sessions and adherence to weekly PA also play crucial roles in achieving desirable outcomes. However, investigating this element in the meta-analysis proved challenging due to the inclusion of various chronic conditions, each with their specific PA guidelines, although they share some similarities.

Moreover, this meta-analysis revealed that resistance training has a large effect on perceived fatigue (SD=0.93) with high heterogeneity and a limited number of studies. Aerobic cycling and combination training were found to have moderate effects on perceived fatigue in adults with chronic conditions (SMD = 0.66, and 0.76, respectively). However, there was variation in heterogeneity among these subgroups. The aerobic cycling training subgroup exhibited no heterogeneity. However, it is important to note that this subgroup had a limited number of studies, which could have influenced the results. In contrast, the combination training subgroup, incorporating different training components such as aerobic, resistance, and balance exercises, displayed high heterogeneity across the studies. Moreover, balance training was found to have a small effect on perceived fatigue (SMD=0.40). The heterogeneity could be attributed by the variations in the combination training programs implemented. Existing literature suggests that aerobic and resistance training have been effective in alleviating fatigue symptoms among individuals with chronic conditions^33,125–127^. Conversely, treadmill running has shown no improvement in fatigue symptoms^119^. Therefore, the literature provides conflicting findings regarding the effects of different PA modes on fatigue, necessitating further exploratory studies in this area. Furthermore, the importance of PA enjoyment has been highlighted in the literature, as it has been observed that individuals who find an activity enjoyable are more likely to stay engaged and experience PA benefits while reducing their fatigue symptoms^50^. Additionally, individuals with chronic disorders may experience discomfort and other symptoms such as shortness of breath during aerobic training; underscoring the need for personalised options tailored to their specific needs. The inclusion of a range of PA modes seems promising, as it allows for choice, but further research is needed in this regard.

The effects of PA interventions on fatigue reduction on post-trial follow up were examined in eight studies included in this review, which revealed small significant effect (SMD=0.38) of the PA intervention on fatigue reduction during the follow up. However, it is important to note that the level of PA participation between the end of the intervention and the follow up period was not clearly outlined in the included studies. Among the two studies that reported guidance during follow up, conflicting approaches were observed. One study motivated participants to maintain an active lifestyle and continue exercising, while another study encouraged them to resume their pre-intervention daily routine to assess the effects of the intervention after a period of inactivity. The contrasting guidance provided to participants could have influenced the effects of PA interventions on fatigue during follow up, as sustained PA during this period might suggest that the effects on fatigue could be diminished in the long term. Furthermore, the scarcity of studies and the heterogeneity preclude definitive conclusions regarding the sustained effect of PA interventions on fatigue reduction. Nevertheless, it was observed that most PA interventions do not include post-trial follow up measurements, which are essential for identifying potential risks that may not be evident during the trial period^128,129^. In many cases, after the standardised intervention length, adults with chronic conditions struggle to remain physically active. Notably, a study aiming to promote long term PA adherence after rehabilitation discharge in individuals with chronic conditions demonstrated successful outcomes even one year after follow-up^130,131^.

Individuals with chronic conditions often experience fatigue, leading to activity avoidance or underactivity^24^. Therefore, it is important to distribute activities throughout the day more effectively^24^. Interestingly, none of the included interventions considered incorporating self-regulatory strategies such as activity pacing (regulation of activity levels) to participants^132^. Integrating activity pacing guidance, taught by healthcare professionals (e.g., occupational or physical therapists) can foster a balanced and active lifestyle, effectively managing fatigue symptoms and promoting physical activity engagement^4,24,133–135^. Moreover, self-regulatory skills have been found important for moderate to vigorous intensity PA and relevant for addressing fatigue complaints^134,136,137^. Thus, self-regulation should be considered in PA interventions, particularly when aiming for sustained physical activity engagement, facilitating the adaption of individuals with fatigue to the new PA lifestyle changes. Overall, the combination of activity pacing and self-regulation holds promise in achieving a physically active lifestyle while effectively managing in the long term^137^. Therefore, it is imperative to explore long term strategies and implement follow up measures that aim to reduce fatigue symptoms among adults with chronic conditions while maintaining PA engagement over time.

Furthermore, our transdiagnostic approach focusing specifically on fatigue symptoms, emphasizes the significance of addressing fatigue symptoms through PA, which can yield numerous benefits. Wilson and Cleary’s health-related quality of life model, highlights the interconnectedness of symptom status, which influences functional health, which influences the general health perception, which consequently influences the overall quality of life^138^. Applying this model to our findings may suggest that improving quality of life in individuals with chronic conditions can be achieved by reducing fatigue symptoms through PA^51^. This perspective could indicate the potential of PA to positively impact the overall well-being and functioning of individuals with chronic conditions. Further research is recommended to provide further evidence on this approach.

### Strengths and limitations

This review possesses several key strengths. It is the first systematic review/meta-analysis to follow a transdiagnostic approach on this specific topic. This approach might enhance the generalizability of the findings and provide important insights into the effects of PA interventions on fatigue reduction across multiple chronic conditions. Additionally, this review synthesises data from thirty-three interventions, allowing for a comprehensive evaluation of the effects and providing a robust foundation for recommendations.

There are also some limitations of this review. Heterogeneity among the studies may limit the generalizability of our findings. Variations in the effects of PA interventions on fatigue may be attributed, in part, to intervention characteristics such as duration, sessions, and mode of PA. Furthermore, fatigue can vary across conditions with different severity levels^139^. Additionally, in the current review, over 65% of the studies were judged as high-risk based on the ROB2 assessment, primarily due to deviations from the intended interventions. Additionally, other elements of PA interventions posed challenges for inclusion in the meta-analysis. For instance, studies reported intensities differently or did not report them at all. Furthermore, the proportion of female participants was higher across the studies compared to males, which could have influenced the results given the higher prevalence of fatigue in females^140–145^.

### Implications

In a transdiagnostic sample, this meta-analysis indicates that PA interventions have a moderate overall effect in reducing perceived fatigue among adults with chronic conditions. This finding indicates the potential integration of PA into fatigue management programs. However, the limited inclusion of follow up measures and long term effects emphasizes the need for further exploration of the sustained impact of PA on fatigue reduction. Additionally, the incorporation of activity pacing and self-regulation into long term interventions is crucial as these strategies have been identified as key factors in PA and fatigue management among adults with chronic conditions. Furthermore, it is important to critically assess and investigate the effectiveness of different ingredients of PA interventions while considering confounding factors, as these outcomes might have significant implications for both healthcare professionals and patients.

## Conclusion

Fatigue poses a significant barrier to PA engagement among adults with chronic conditions, but our findings provide robust evidence supporting the moderate effects of PA in reducing fatigue in this population. Our meta-analysis revealed that resistance, aerobic cycling, combination, and balance training interventions demonstrated significant effects on fatigue reduction. Notably, both short (2-6 weeks) and longer-term (16-24 weeks) interventions had a large effect in reducing fatigue. However, the observed effects on fatigue during post-trial follow-ups were small, due to the lack of studies conducting follow up measurements underscoring the importance of further investigation into the long-term effects of PA interventions. Additionally, further research is needed on the effects of the specific intervention ingredients as these findings hold valuable implications for health professionals and patients.

## Data Availability

All data generated or analysed during this meta-analysis review are included in this published article. There was no patient and public involvement in this study. Dissemination to study participants is not applicable.

## Abbreviations

BMI: Body mass index
M: Mean
SD: Standard deviation
SMD: Standardised mean difference
CI: Confidence intervals
RCTs: Randomised controlled trials
PA: Physical activity
MeSH: Medical Subject Headings
MS: Multiple sclerosis
BC: Breast cancer
COPD: Chronic obstructive pulmonary disease
FMS: Fibromyalgia syndrome
CC: Colorectal cancer
C: Cancer
KF: Kidney failure
PD: Parkinson’s disease
IBS: Irritable bowel syndrome
RA: Rheumatoid arthritis
AS: Axial spondylarthritis
FSS: Fatigue severity scale
FACIT-F: Functional assessment of chronic illness therapy—fatigue
FAQ: Fatigue assessment questionnaire
MFIS: Modified fatigue impact scale
MFI: Multidimensional fatigue inventory
VAS: Visual analogue scale
IG: Intervention group
EG: Exercise group
RC: Relaxation group
CG: Control group
MHR: Maximum heart rate
RPM: Revolutions per minute
APMHR: Age predicted maximum heart rate
mins: Minutes
MET: Metabolic equivalent of task
RM: Repetition maximum
RPM: Revolutions per minute
THR: Target heart rate
HRR: Heart rate reserve
ES: Effect size
TOCT: Task-oriented circuit training
AGU: Augmented Reality Applications in Rehabilitation
IB: Ioulia Barakou
KES: Kandianos Emmanouil Sakalidis
USA: Ulric Sena Abonie
FJH: Florentina Johanna Hettinga
