# Author Correction: Effectiveness of physical activity interventions on reducing perceived fatigue among adults with chronic conditions: a systematic review and meta-analysis of randomised controlled trials

**DOI:** 10.1038/s41598-024-77454-y

**Published:** 2025-01-22

**Authors:** Ioulia Barakou, Kandianos Emmanouil Sakalidis, Ulric Sena Abonie, Tracy Finch, Katie L. Hackett, Florentina Johanna Hettinga

**Affiliations:** 1 Department of Nursing, Midwifery & Health, Northumbria University, Newcastle Upon Tyne, NE7 7XA UK; 2 Department of Sport Exercise and Rehabilitation, Faculty of Health and Life Sciences, Northumberland Building, Northumbria University, Newcastle Upon Tyne, NE1 8ST UK; 3 Department of Social Work, Education and Community Wellbeing, Northumbria University, Newcastle Upon Tyne, UK; 4 CRESTA Fatigue Clinic, Newcastle upon Tyne Hospitals NHS Foundation Trust, Newcastle Upon Tyne, UK

Correction to: *Scientific Reports* 10.1038/s41598-023-41075-8, published online 04 September 2023

The original version of this Article contained an error in the meta-analysis, resulting in corrections in the Abstract, the Results section, the Discussion section, the Conclusion, in Figures 3, 4, 5, 6, 7 and 8, as well as the Reference list. Due to a discrepancy in the meta-analysis, some of the meta-analysis scores were analyzed inaccurately. More specifically, a positive effect of some interventions using the FACIT questionnaire was mistaken for a negative effect in the meta-analysis.

In the Abstract,

“Overall, physical activity interventions moderately reduced fatigue (SMD = 0.54, *p* < 0.0001). Interventions lasting 2–6 weeks demonstrated a larger effect on fatigue reduction (SMD = 0.86, *p* < 0.00001). Interventions with 18–24 sessions showed a large effect on fatigue reduction (SMD = 0.97, *p* < 0.00001). Aerobic cycling and combination training interventions had a large to moderate effect (SMD = 0.66, *p* = 0.0005; SMD = 0.60, *p* = 0.0010, respectively). No long-term effects were found during follow-up.”

now reads:

“Overall, physical activity interventions moderately reduced fatigue (SMD = 0.70, *p* < 0.0001). Interventions lasting 2–6 weeks and 16–24 weeks demonstrated the larger effects on fatigue reductions (SMD = 0.86, *p* < 0.00001; SMD = 1.82, *p* = 0.01, respectively). Interventions with 30–36 sessions showed a large effect on fatigue reduction (SMD  = 0.94, *p* = 0.04). Resistance, aerobic cycling and combination training interventions had a large to moderate effect (SMD = 0.93, *p* = 0.03; SMD = 0.66, *p* = 0.0005; SMD = 0.76, *p* = < 0.00001, respectively). Small long-term effects were found during follow-up (SMD = 0.38, *p* = 0.002). Notably, both short (2–6 weeks) and longer-term (16–24 weeks) interventions were effective in reducing fatigue.”

In the Results section, under the subheading ‘Effects of PA interventions on perceived fatigue’,

“Initially, a meta-analysis was conducted to investigate the effects of all the included PA interventions on perceived fatigue. Eventually, 31 articles out of the 38 were included in the meta-analysis. The test for overall effect indicates that there is a moderate effect for reduction in perceived fatigue based on random effects model (SMD = 0.54; 95% CI = 0.79 to 0.29; *p* < 0.00001) with high heterogeneity results between studies (I^2^ = 79%). The outcomes are illustrated in Fig. 3.

The effects of the intervention length on perceived fatigue are presented in Fig. 4. The test for subgroup differences was not statistically significant with low heterogeneity (*p* = 0.38, I^2^ = 2.5%). Interventions that lasted for 2–6 weeks have a larger effect (SMD = 0.86; 95% CI = 1.24 to 0.48; *p* < 0.00001) compared to interventions that were 7–10 weeks (SMD = 0.76; 95% CI = 1.22 to 0.29; *p* = 0.001). The results from the interventions that were 2–6 weeks showed low heterogeneity (I^2^ = 10%) while the results from the interventions that were 7–10 weeks showed high heterogeneity (I^2^ = 79%). Moreover, the interventions that were 11–15 weeks showed a low effect for perceived fatigue (SMD = 0.48; 95% CI = 0.76–0.20; *p* = 0.0008) and results were heterogeneous between studies (I^2^ = 72%). No effect was found for the interventions that were 16 weeks + (SMD = 0.07; 95% CI = 1.86–1.73; *p* = 0.94) and results were heterogeneous between studies (I^2^ = 93%).

The effects of the intervention total sessions of PA interventions on perceived fatigue are illustrated in Fig. 5. The test for the subgroup differences was statistically significant with high heterogeneity (*p* = 0.01; I^2^ = 68%). Meta-analysis showed no effect in perceived fatigue in the 8–16 sessions (SMD = 0.23; 95% CI = 0.59–0.14; *p* = 0.23) and results were heterogeneous between studies (I^2^ = 51%). Interventions with 18–24 sessions had a large effect for perceived fatigue (SMD =  −0.97; 95% CI =  −1.29 to −0.64; *p* < 0.00001) and results were found heterogenous (I^2^ = 70%). The interventions with 30–36 sessions showed no effect (SMD = 0.21; 95% CI = 1.16–0.75; *p* = 0.67) and heterogeneity was found between studies (I^2^ = 91%). Likewise, meta-analysis showed no effect for perceived fatigue in the interventions of 45–48 sessions (SMD = 0.22; 95% CI = 0.72–1.17; *p* = 0.64) and results between the studies were heterogeneous (I^2^ = 85%). Similarly, interventions with 54 + sessions were found to have no statistically significant results (SMD = 0.79; 95% CI =  −2.00 to 0.41; *p* = 0.20, I^2^ = 89%).

The effects of the mode of PA interventions on perceived fatigue are illustrated in Fig. 6. Overall, the test for subgroup differences suggests that there is a statistically significant subgroup effect based on a random model with high heterogeneity (*p* = 0.002; I^2^ = 71.2%). Meta-analysis showed no effect for perceived fatigue in the aerobic running training subgroup (SMD = 1.25; 95% CI =  −3.61 to 1.11; *p* = 0.30) and results between studies were heterogenous (I^2^ = 89%). The estimates showed a moderate effect for perceived fatigue in the aerobic cycling training interventions (SMD =  −0.66; 95% CI =  −1.03 to −0.29; *p* = 0.0005) and the results between studies were not heterogeneous (I^2^ = 0%). Interventions with balance training showed a low effect for perceived fatigue (SMD =  −0.40; 95% CI =  −0.80 to −0.01; *p* < 0.0001) and results between studies were moderately heterogeneous (I^2^ = 60%). Interventions with resistance training showed no statistically significant results for perceived fatigue (SMD = 0.54; 95% CI = 1.43–0.35; *p* = 0.24, I^2^ = 86%). Interventions with combination training showed a moderate effect for perceived fatigue (SMD = 0.60; 95% CI = 0.95–0.24; *p* = 0.0010) and results were heterogeneous between studies (I^2^ = 80%). Interventions with aerobic exergaming and horseback riding were not included in the subgroup meta-analysis since there was only one study in each group.

A meta-analysis was conducted for the post-trial follow ups. There were only eight studies that included post-trial follow up measurements. The test for the long-term effects showed no effect for perceived fatigue (SMD = 0.32; 95% CI = 0.60–0.04; *p* = 0.003) and results were moderately heterogenous between studies (I^2^ = 45%). The outcomes are illustrated in Fig. 7.”

now reads:

“Initially, a meta-analysis was conducted to investigate the effects of all the included PA interventions on perceived fatigue. Eventually, 31 articles out of the 38 were included in the meta-analysis. The test for overall effect indicates that there is a moderate effect for reduction in perceived fatigue based on random effects model (SMD = 0.70; 95% CI = 0.94 to 0.47; *p* < 0.00001) with high heterogeneity results between studies (I^2^ = 77%). The outcomes are illustrated in Fig. 3.

The effects of the intervention length on perceived fatigue are presented in Fig. 4. The test for subgroup differences was not statistically significant with moderate heterogeneity (*p* = 0.17, I^2^ = 40.9%). Interventions that lasted for 2–6 weeks have a larger effect (SMD = 0.86; 95% CI = 1.24 to 0.48; *p* < 0.00001) compared to interventions that were 7–10 weeks (SMD = 0.77; 95% CI = 1.27 to 0.26; *p* = 0.003). The results from the interventions that were 2–6 weeks showed low heterogeneity (I^2^ = 10%) while the results from the interventions that were 7–10 weeks showed high heterogeneity (I^2^ = 82%). Moreover, the interventions that were 11–15 weeks showed a low effect for perceived fatigue (SMD = 0.49; 95% CI = 0.77 to −0.21; *p* = 0.0005) and results were heterogeneous between studies (I^2^ = 71%). A high effect was found for the interventions that were 16 weeks + (SMD = 1.82; 95% CI = −3.28 to −0.36; *p* = 0.01) and results were heterogeneous between studies (I2 = 88%).

The effects of the intervention total sessions of PA interventions on perceived fatigue are illustrated in Fig. 5. The test for the subgroup differences was not statistically significant with low heterogeneity (*p* = 0.29; I^2^ = 18.9%). Meta-analysis showed a low effect on perceived fatigue in the 8–16 sessions (SMD = 0.38; 95% CI = −0.69 to −0.07; *p* = 0.02) and results were moderately heterogeneous between studies (I^2^ = 33%). Interventions with 18–24 sessions had a large effect for perceived fatigue (SMD = 0.87; 95% CI =  −1.26 to −0.47; *p* < 0.0001) and results were found heterogenous (I^2^ = 80%). The interventions with 30–36 sessions showed a high effect (SMD = 0.94; 95% CI = −1.58 to −0.31; *p* = 0.004) and heterogeneity was found between studies (I^2^ = 79%). Inversely, meta-analysis showed no effect for perceived fatigue in the interventions of 45–48 sessions (SMD = 0.64; 95% CI = −1.32 to −0.05; *p* = 0.07) and results between the studies were heterogeneous (I^2^ = 70%). Similarly, interventions with 54 + sessions were found to have no statistically significant results (SMD = 0.80; 95% CI =  −2.02 to 0.38; *p* = 0.18, I^2^ = 89%).

The effects of the mode of PA interventions on perceived fatigue are illustrated in Fig. 6. Overall, the test for subgroup differences suggests that there is a statistically significant subgroup effect based on a random model with high heterogeneity (*p* = 0.0001; I^2^ = 77.8%). Meta-analysis showed no effect for perceived fatigue in the aerobic running training subgroup (SMD = 1.25; 95% CI =  −3.61 to 1.11; *p* = 0.30) and results between studies were heterogenous (I^2^ = 89%). The estimates showed a moderate effect for perceived fatigue in the aerobic cycling training interventions (SMD =  −0.66; 95% CI =  −1.03 to −0.29; *p* = 0.0005) and the results between studies were not heterogeneous (I^2^ = 0%). Interventions with balance training showed a low effect for perceived fatigue (SMD =  −0.40; 95% CI =  −0.80 to −0.01; *p* = 0.05) and results between studies were moderately heterogeneous (I^2^ = 60%). Interventions with resistance training showed a high effect for perceived fatigue (SMD = 0.93; 95% CI = −1.75 to −0.12; *p* = 0.03, I^2^ = 83%). Interventions with combination training showed a moderate effect for perceived fatigue (SMD = 0.76; 95% CI = −1.07 to −0.45; *p* < 0.00001) and results were heterogeneous between studies (I^2^ = 74%). Interventions with aerobic exergaming and horseback riding were not included in the subgroup meta-analysis since there was only one study in each group.

A meta-analysis was conducted for the post-trial follow ups. There were only eight studies that included post-trial follow up measurements. The test for the long-term effects showed a low effect for perceived fatigue (SMD = 0.38; 95% CI = −0.62 to −0.14; *p* = 0.002) and results were moderately heterogenous between studies (I^2^ = 29%). The outcomes are illustrated in Fig. 7.”

In addition, under the subheading ‘Publication bias’,

“The publication bias assessment was estimated on the meta-analysis data. The analysis showed statistically significant evidence of publication bias (z = −4.3163, *p* < 0.0001). The funnel plot demonstrated visible asymmetry, indicating the possibility of publication bias. The estimated intercept of the regression line was −0.7805 (95% CI: −1.0314 to −0.5296) when the standard error of the effect sizes approached zero. The funnel plot is illustrated in Fig. 8.”

now reads:

“The publication bias assessment was estimated on the meta-analysis data. The analysis showed statistically significant evidence of publication bias (z = −2.77, *p* = 0.006). The funnel plot demonstrated visible asymmetry, indicating the possibility of publication bias. The estimated intercept of the regression line was −1.38 (95% CI: −5.45 to 2.68) when the standard error of the effect sizes approached zero. The funnel plot is illustrated in Fig. 8.”

Further, in the Discussion section,

“Firstly, this meta-analysis demonstrates that PA interventions have a moderate effect on perceived fatigue (SMD = 0.54) among adults with chronic conditions.”

now reads:

“Firstly, this meta-analysis demonstrates that PA interventions have a moderate effect on perceived fatigue (SMD = 0.70) among adults with chronic conditions.”

And,

“Regarding the intervention length, this meta-analysis revealed that trials lasting from two to ten weeks^2,3,4,5,6,7,8,9,10^ demonstrated a large to moderate effect on perceived fatigue among adults with chronic conditions (SMD = 0.86, 0.76, respectively). Additionally, the subgroup of eleven to fifteen showed a low to moderate effect (SMD = 0.48) However, high heterogeneity was observed in the seven to ten weeks and eleven to fifteen weeks subgroups, potentially influenced by other intervention characteristics such as total sessions or mode of PA or even the disease diagnosis. In contrast, low heterogeneity was found in the 2–6 weeks intervention length, although the limited number of trials in this subgroup may be a limitation. Trials lasting from sixteen to 24 weeks were found to have no effect on perceived fatigue, with high heterogeneity and limited trials in this subgroup posing potential limitations. Additionally, adherence to the intervention was reported in two out of the four trials in this subgroup^71,80,81,92^, with adherence rates of 65%^92^ and 93.3%^71^. Due to lack of adherence reporting, we cannot definitively conclude that adherence might be a reason for the low effect on perceived fatigue. The duration of an intervention holds importance for health professionals and researchers, as clinical and health interventions often face budget constraints^122^. While this meta-analysis suggests a trend of shorter PA interventions being effective for alleviating fatigue, other factors may influence the results. Therefore, future research is necessary to determine the optimal and effective intervention duration, considering factors such as cost-effectiveness and time-efficiency in research and rehabilitation settings.

The exploration of total sessions in PA interventions yielded interesting findings. It was observed that interventions comprising 18–24 sessions had a substantial impact on perceived fatigue (SMD = 0.97). The considerable heterogeneity observed in the results could be partially attributed to other intervention characteristics, such as intervention duration and mode. On the other hand, subgroups with 8–16, 30–36, 45–48, and 54 + total sessions showed no effect on perceived fatigue. The uneven distribution of studies among these subgroups might have limited the analysis of their effects on the total sessions of the interventions. Thus, researchers should be careful when interpreting the pooled effect sizes and focus on the observed data patterns. Furthermore, the duration of PA sessions and adherence to weekly PA also play crucial roles in achieving desirable outcomes. However, investigating this element in the meta-analysis proved challenging due to the inclusion of various chronic conditions, each with their specific PA guidelines, although they share some similarities.

Moreover, this meta-analysis revealed that aerobic cycling and combination training have moderate effects on perceived fatigue in adults with chronic conditions (SMD = 0.66, and 0.60, respectively). However, there was variation in heterogeneity among these subgroups. The aerobic cycling training subgroup exhibited no heterogeneity. However, it is important to note that this subgroup had a limited number of studies, which could have influenced the results. In contrast, the combination training subgroup, incorporating different training components such as aerobic, resistance, and balance exercises, displayed high heterogeneity across the studies. The heterogeneity could be attributed by the variations in the combination training programs implemented. Existing literature suggests that aerobic and resistance training have been effective in alleviating fatigue symptoms among individuals with chronic conditions^33,123,124,125^. Conversely, treadmill running has shown no improvement in fatigue symptoms^119^. Therefore, the literature provides conflicting findings regarding the effects of different PA modes on fatigue, necessitating further exploratory studies in this area. Furthermore, the importance of PA enjoyment has been highlighted in the literature, as it has been observed that individuals who find an activity enjoyable are more likely to stay engaged and experience PA benefits while reducing their fatigue symptoms^50^. Additionally, individuals with chronic disorders may experience discomfort and other symptoms such as shortness of breath during aerobic training; underscoring the need for personalised options tailored to their specific needs. The inclusion of a range of PA modes seems promising, as it allows for choice, but further research is needed in this regard.

The effects of PA interventions on fatigue reduction on post-trial follow up were examined in eight studies included in this review, which revealed no significant effect of the PA intervention on fatigue reduction during the follow up. However, it is important to note that the level of PA participation between the end of the intervention and the follow up period was not clearly outlined in the included studies. Among the two studies that reported guidance during follow up, conflicting approaches were observed. One study motivated participants to maintain an active lifestyle and continue exercising, while another study encouraged them to resume their pre-intervention daily routine to assess the effects of the intervention after a period of inactivity. The contrasting guidance provided to participants could have influenced the effects of PA interventions on fatigue during follow up, as sustained PA during this period might suggest that the effects on fatigue could be diminished in the long term. Furthermore, the scarcity of studies and the high heterogeneity preclude definitive conclusions regarding the sustained effect of PA interventions on fatigue reduction. Nevertheless, it was observed that most PA interventions do not include post-trial follow up measurements, which are essential for identifying potential risks that may not be evident during the trial period^126,127^. In many cases, after the standardised intervention length, adults with chronic conditions struggle to remain physically active. Notably, a study aiming to promote long term PA adherence after rehabilitation discharge in individuals with chronic conditions demonstrated successful outcomes even one year after follow-up^128,129^.”

now reads:

“Regarding the intervention length, this meta-analysis revealed that trials lasting from two to six weeks and sixteen to twenty-four weeks showed large effects on reducing perceived fatigue among adults with chronic conditions (SMD = 0.86, 1.82, respectively). Low heterogeneity was found in two to six weeks intervention length, although the limited number of trials in this subgroup may be a limitation. High heterogeneity was found in the sixteen to twenty-four weeks subgroup, while limited trials in this subgroup could pose potential limitations. Additionally, adherence to the intervention was reported in two out of the four trials in the sixteen to twenty-four weeks subgroup^71,80,81,92^, with adherence rates of 65%^92^ and 93.3%^71^. The short-term interventions may benefit from immediate results as it can be something new to the participants, leading to a rapid reduction in the perceived fatigue. While longer interventions may result in more sustained changes over time, hence the larger effect in perceived fatigue. In literature, longer interventions (6–12 months) have been found to be effective in achieving behavior change for example in nutrition and PA^122,123^. However, in this study due to the limited number of trials in these subgroups, we cannot draw any conclusions. Additionally, seven to ten weeks demonstrated a moderate effect on perceived fatigue among adults with chronic conditions (SMD = 0.77). The subgroup of eleven to fifteen weeks showed a moderate effect (SMD = 0.49). However, high heterogeneity was observed in these two subgroups, potentially influenced by other intervention characteristics such as total sessions or mode of PA or even the disease diagnosis. The duration of an intervention holds importance for health professionals and researchers, as clinical and health interventions often face budget constraints^124^. While this meta-analysis suggests a trend of shorter PA interventions (up to 24 weeks) being effective for reducing fatigue, other factors may influence the results. Therefore, future research is necessary to determine the optimal and effective intervention duration, considering factors such as cost-effectiveness and time-efficiency in research and rehabilitation settings.

The exploration of total sessions in PA interventions yielded interesting findings. It was observed that interventions comprising 18–24 and 30–36 sessions had a substantial impact on perceived fatigue (SMD = 0.87 and 0.94, respectively). The considerable heterogeneity observed in the results could be partially attributed to other intervention characteristics, such as intervention duration and mode. Moreover, interventions comprising 8–16 weeks have a low impact on perceived fatigue (SMD = 0.38). On the other hand, subgroups with 45–48, and 54 + total sessions showed no effect on perceived fatigue. The uneven distribution of studies among these subgroups might have limited the analysis of their effects on the total sessions of the interventions. Thus, researchers should be careful when interpreting the pooled effect sizes and focus on the observed data patterns. Furthermore, the duration of PA sessions and adherence to weekly PA also play crucial roles in achieving desirable outcomes. However, investigating this element in the meta-analysis proved challenging due to the inclusion of various chronic conditions, each with their specific PA guidelines, although they share some similarities.

Moreover, this meta-analysis revealed that resistance training has a large effect on perceived fatigue (SD = 0.93) with high heterogeneity and a limited number of studies. Aerobic cycling and combination training were found to have moderate effects on perceived fatigue in adults with chronic conditions (SMD = 0.66, and 0.76, respectively). However, there was variation in heterogeneity among these subgroups. The aerobic cycling training subgroup exhibited no heterogeneity. However, it is important to note that this subgroup had a limited number of studies, which could have influenced the results. In contrast, the combination training subgroup, incorporating different training components such as aerobic, resistance, and balance exercises, displayed high heterogeneity across the studies. Moreover, balance training was found to have a small effect on perceived fatigue (SMD = 0.40). The heterogeneity could be attributed by the variations in the combination training programs implemented. Existing literature suggests that aerobic and resistance training have been effective in alleviating fatigue symptoms among individuals with chronic conditions^33,125,126,127^. Conversely, treadmill running has shown no improvement in fatigue symptoms^119^. Therefore, the literature provides conflicting findings regarding the effects of different PA modes on fatigue, necessitating further exploratory studies in this area. Furthermore, the importance of PA enjoyment has been highlighted in the literature, as it has been observed that individuals who find an activity enjoyable are more likely to stay engaged and experience PA benefits while reducing their fatigue symptoms^50^. Additionally, individuals with chronic disorders may experience discomfort and other symptoms such as shortness of breath during aerobic training; underscoring the need for personalised options tailored to their specific needs. The inclusion of a range of PA modes seems promising, as it allows for choice, but further research is needed in this regard.

The effects of PA interventions on fatigue reduction on post-trial follow up were examined in eight studies included in this review, which revealed small significant effect (SMD = 0.38) of the PA intervention on fatigue reduction during the follow up. However, it is important to note that the level of PA participation between the end of the intervention and the follow up period was not clearly outlined in the included studies. Among the two studies that reported guidance during follow up, conflicting approaches were observed. One study motivated participants to maintain an active lifestyle and continue exercising, while another study encouraged them to resume their pre-intervention daily routine to assess the effects of the intervention after a period of inactivity. The contrasting guidance provided to participants could have influenced the effects of PA interventions on fatigue during follow up, as sustained PA during this period might suggest that the effects on fatigue could be diminished in the long term. Furthermore, the scarcity of studies and the heterogeneity preclude definitive conclusions regarding the sustained effect of PA interventions on fatigue reduction. Nevertheless, it was observed that most PA interventions do not include post-trial follow up measurements, which are essential for identifying potential risks that may not be evident during the trial period^128,129^. In many cases, after the standardised intervention length, adults with chronic conditions struggle to remain physically active. Notably, a study aiming to promote long term PA adherence after rehabilitation discharge in individuals with chronic conditions demonstrated successful outcomes even one year after follow-up^130,131^.”

Finally, in the Conclusion,

“Fatigue poses a significant barrier to PA engagement among adults with chronic conditions but our findings provide robust evidence supporting the moderate effects of PA in reducing fatigue in this population. Our meta-analysis revealed that both aerobic cycling and combination training interventions demonstrated moderate effects on fatigue reduction. Furthermore, interventions lasting 2–10 weeks showed promising results in reducing fatigue in chronic conditions. However, the observed effects on fatigue during post-trial follow-ups were low, due to the lack of studies conducting follow up measurements underscoring the importance of further investigation into the long term effects of PA interventions. Additionally, further research is needed on the effects of the specific intervention ingredients as these findings hold valuable implications for health professionals and patients.”

now reads:

“Fatigue poses a significant barrier to PA engagement among adults with chronic conditions, but our findings provide robust evidence supporting the moderate effects of PA in reducing fatigue in this population. Our meta-analysis revealed that resistance, aerobic cycling, combination, and balance training interventions demonstrated significant effects on fatigue reduction. Notably, both short (2–6 weeks) and longer-term (16–24 weeks) interventions had a large effect in reducing fatigue. However, the observed effects on fatigue during post-trial follow-ups were small, due to the lack of studies conducting follow up measurements underscoring the importance of further investigation into the long-term effects of PA interventions. Additionally, further research is needed on the effects of the specific intervention ingredients as these findings hold valuable implications for health professionals and patients.”

As a result of the discrepancy in the meta-analysis the data in Figures 3, 4, 5, 6, 7 and 8 have been corrected.

The original Figures and accompanying legends appear below.

**Fig. 3:**
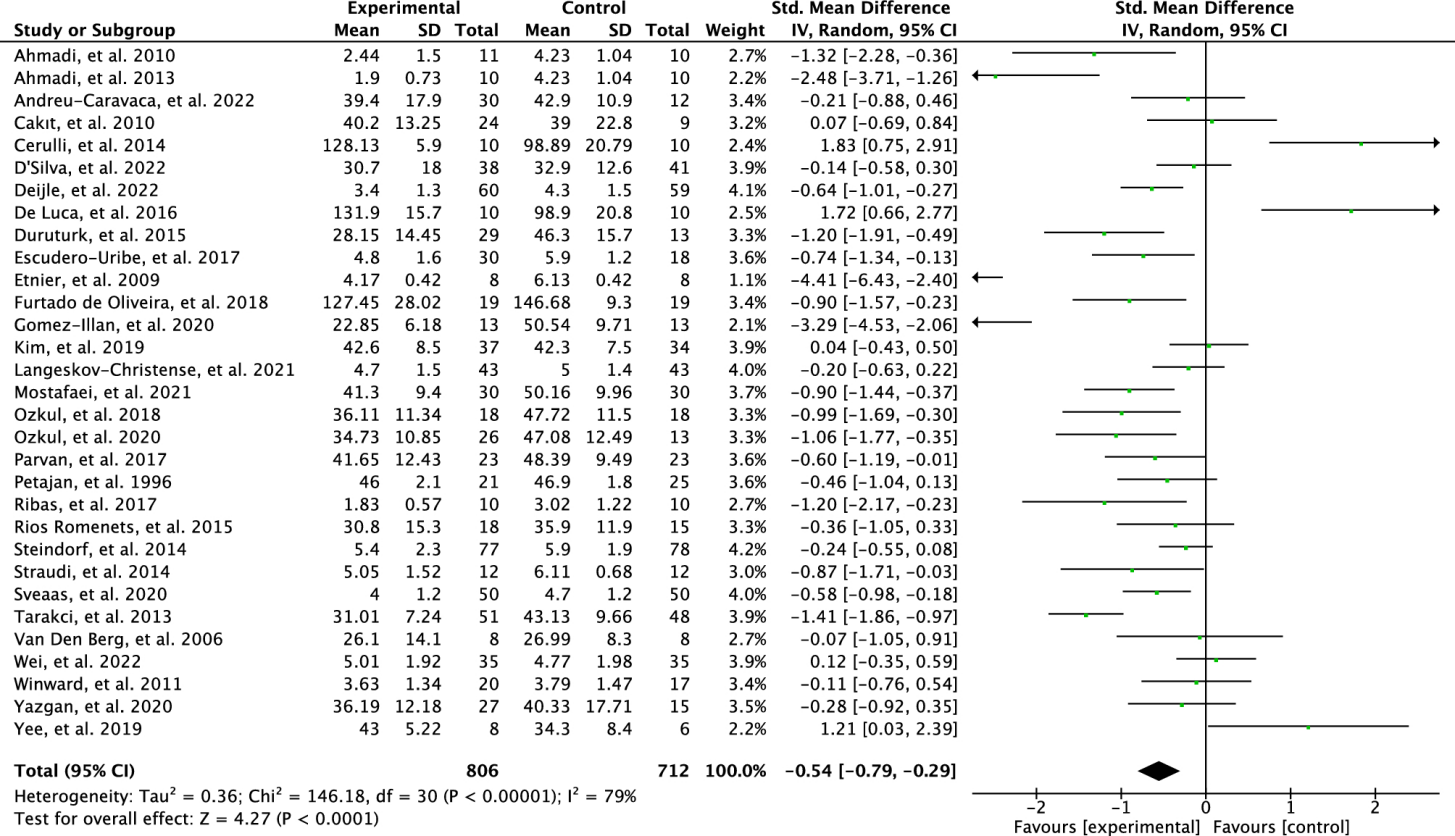
Forest plot of effects of physical activity interventions on perceived fatigue.

**Fig. 4:**
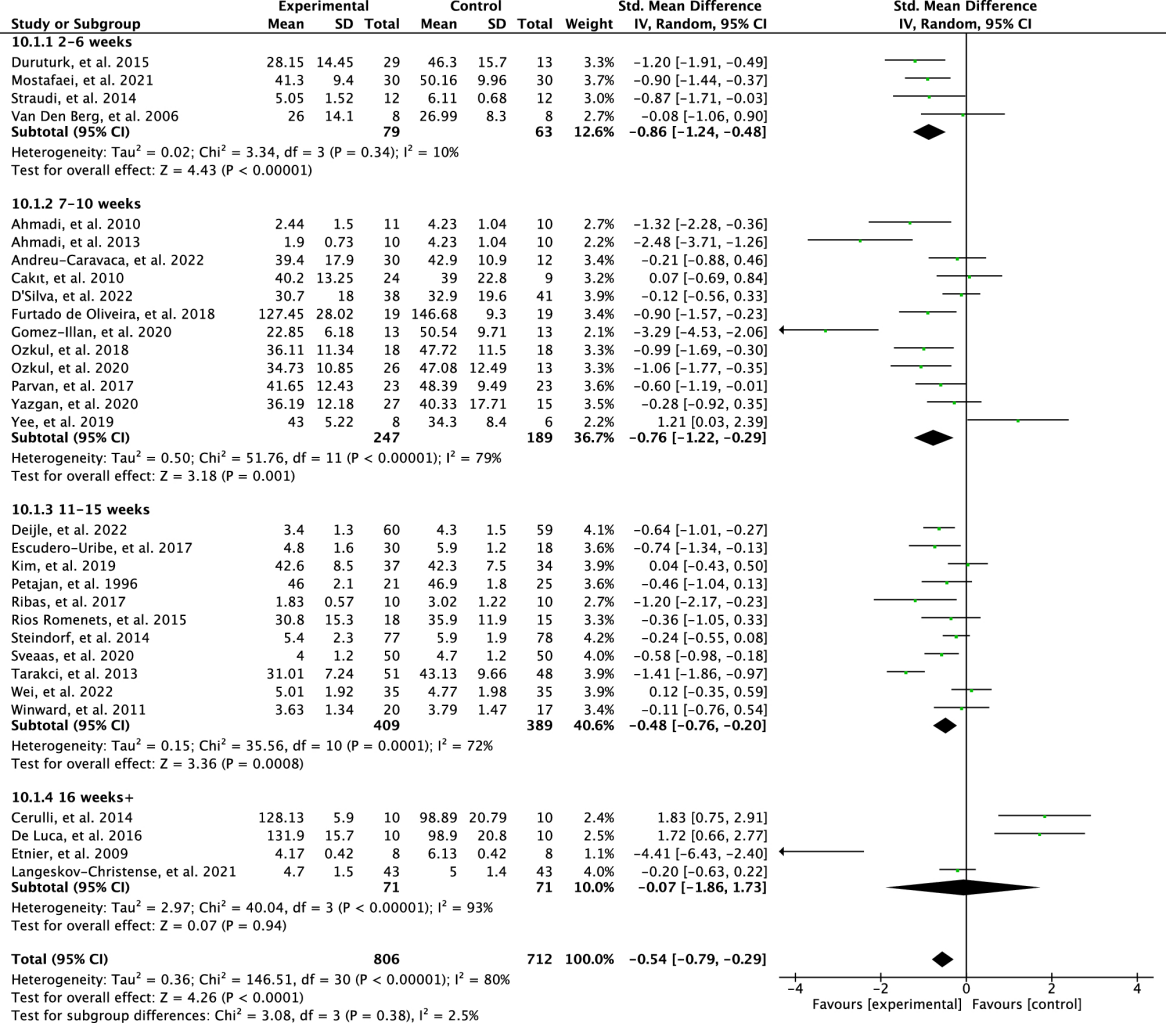
Forest plot of effects of physical activity interventions length on perceived fatigue.

**Fig. 5:**
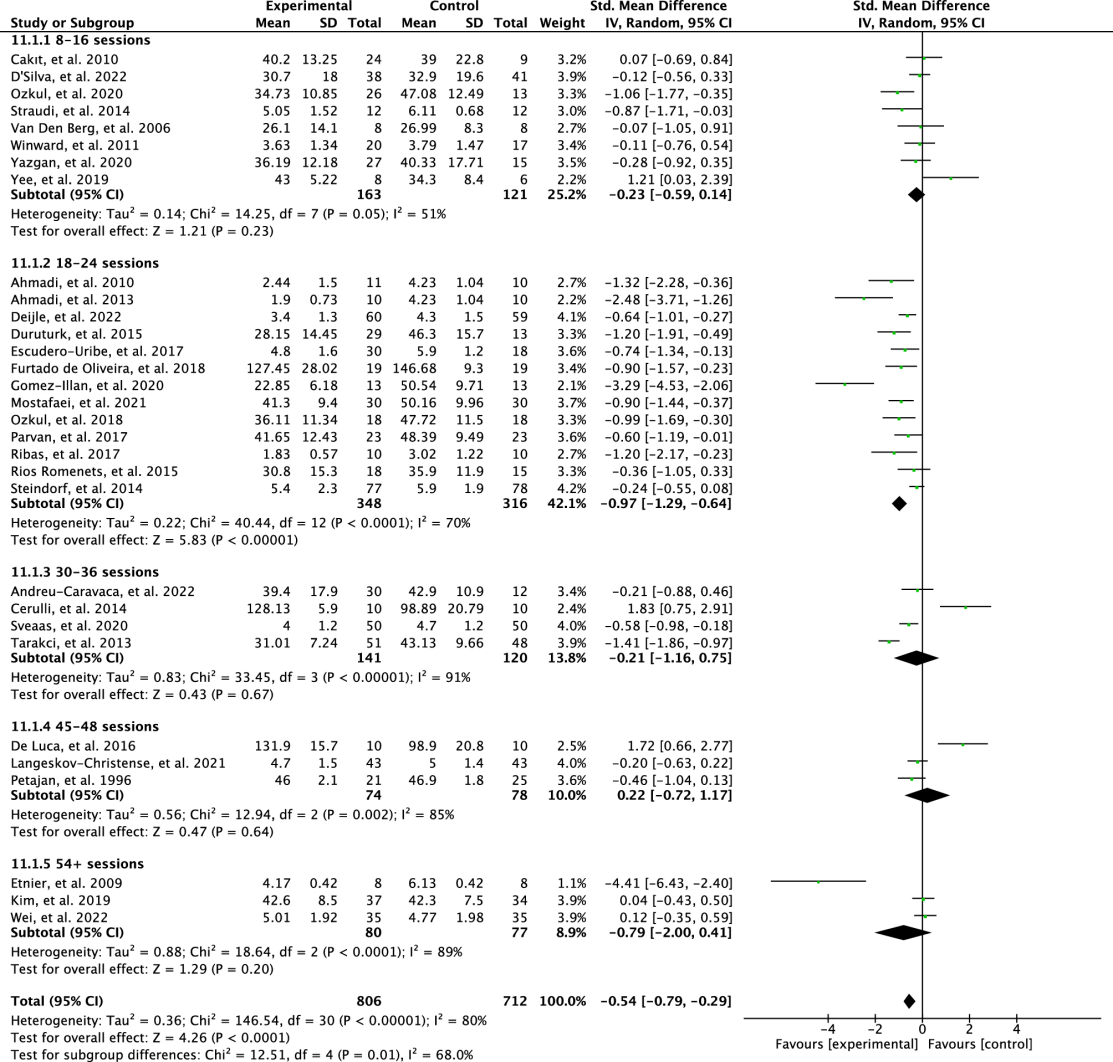
Forest plot of effects of total sessions of physical activity interventions on perceived fatigue.

**Fig. 6:**
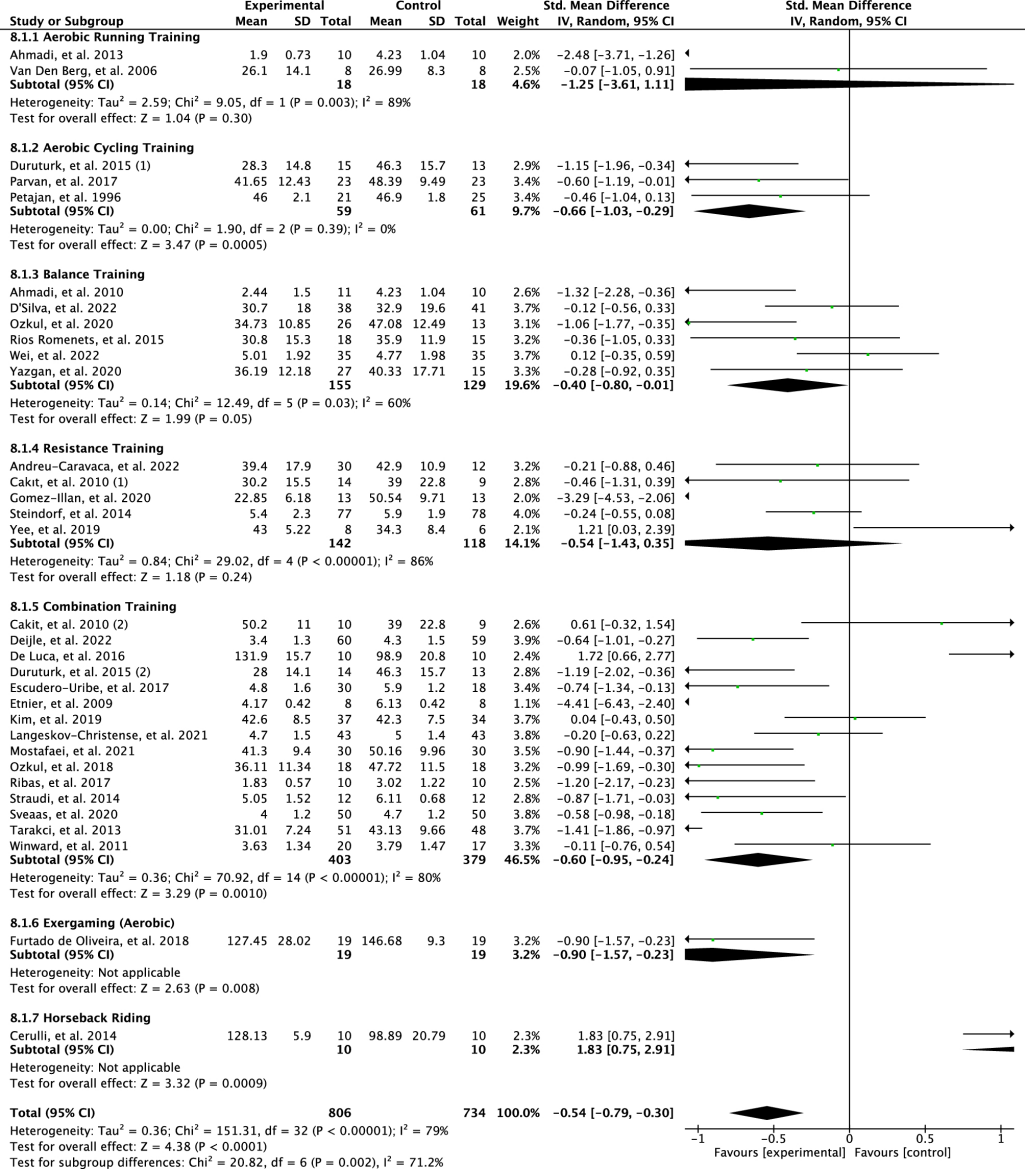
Forest plot of effects of modes of physical activity interventions on perceived fatigue.

**Fig. 7:**
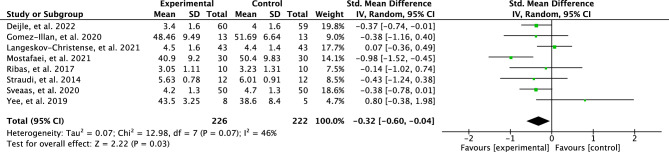
Forest plot of effects of post physical activity interventions follow up.

**Fig. 8:**
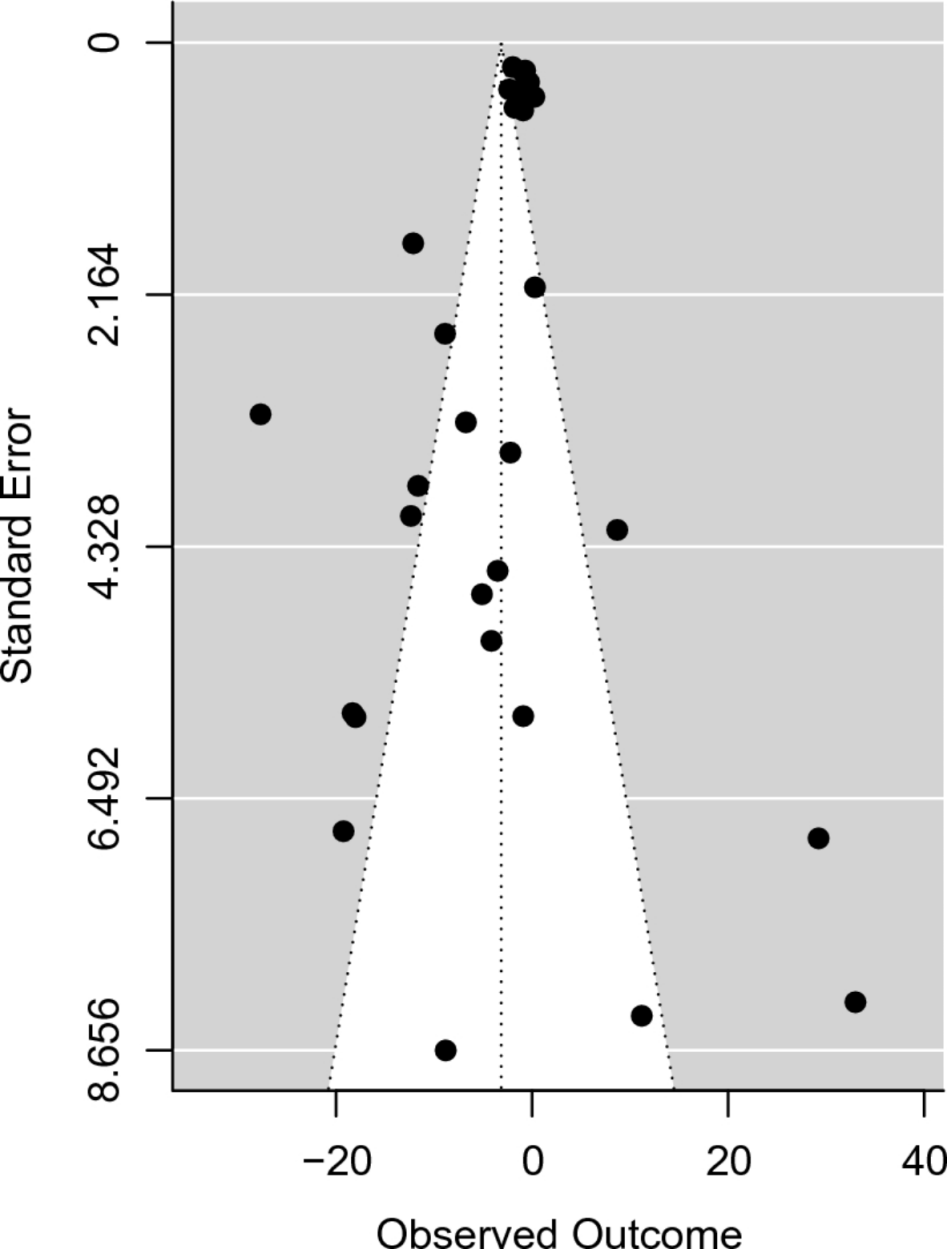
Funnel plot of publication bias on the meta-analysis data.

Additionally, References 122 and 123 were added and are listed below.

122. Arnason A., Langarica N., Dugas L.R., Mora N., Luke A., Markossian T. Family-based lifestyle interventions: What makes them successful? A systematic literature review. *Prev Med Rep*. **21**, 101299. 10.1016/j.pmedr.2020.101299 (2021).

123. Tariq, M.N.M., Stojanovska, L., Dhaheri, A.S.A., Cheikh Ismail, L., Apostolopoulos, V., Ali, H.I. Lifestyle Interventions for Prevention and Management of Diet-Linked Non-Communicable Diseases among Adults in Arab Countries. *Healthcare.* **11**(1), 45. 10.3390/healthcare11010045 (2023).

The subsequent References have been renumbered in the text and in the Reference list.

The original Article has been corrected.

